# Circulating levels of micronutrients and risk of infections: a Mendelian randomization study

**DOI:** 10.1186/s12916-023-02780-3

**Published:** 2023-03-08

**Authors:** Helene M. Flatby, Anuradha Ravi, Jan K. Damås, Erik Solligård, Tormod Rogne

**Affiliations:** 1 Gemini Center for Sepsis Research, Department of Circulation and Medical Imaging, NTNU, Norwegian University of Science and Technology, Prinsesse Kristinas gate 3, Akutten og Hjerte-lunge-senteret, 3. etg, 7491 Trondheim, Norway; 2 Clinic of Anaesthesia and Intensive Care, St. Olavs Hospital, Trondheim University Hospital, Trondheim, Norway; 3 Centre of Molecular Inflammation Research, Department of Clinical and Molecular Medicine, NTNU, Norwegian University of Science and Technology, Trondheim, Norway; 4 Department of Infectious Diseases, St. Olavs Hospital, Trondheim University Hospital, Trondheim, Norway; 5 Department of Chronic Disease Epidemiology and Center for Perinatal, Pediatric and Environmental Epidemiology, Yale School of Public Health, New Haven, CT USA

**Keywords:** Micronutrients, Mendelian randomization, Gastrointestinal infections, Infections, Copper

## Abstract

**Background:**

Micronutrients play an essential role at every stage of the immune response, and deficiencies can therefore lead to increased susceptibility to infections. Previous observational studies and randomized controlled trials of micronutrients and infections are limited. We performed Mendelian randomization (MR) analyses to evaluate the effect of blood levels of eight micronutrients (copper, iron, selenium, zinc, beta-carotene, vitamin B12, vitamin C, and vitamin D) on the risk of three infections (gastrointestinal infections, pneumonia, and urinary tract infections).

**Methods:**

Two-sample MR was conducted using publicly available summary statistics from independent cohorts of European ancestry. For the three infections, we used data from UK Biobank and FinnGen. Inverse variance-weighted MR analyses were performed, together with a range of sensitivity analyses. The threshold for statistical significance was set at *P* < 2.08E−03.

**Results:**

We found a significant association between circulating levels of copper and risk of gastrointestinal infections, where a one standard deviation increase in blood levels of copper was associated with an odds ratio of gastrointestinal infections of 0.91 (95% confidence interval 0.87 to 0.97, *P* = 1.38E−03). This finding was robust in extensive sensitivity analyses. There was no clear association between the other micronutrients and the risk of infection.

**Conclusions:**

Our results strongly support a role of copper in the susceptibility to gastrointestinal infections.

**Supplementary Information:**

The online version contains supplementary material available at 10.1186/s12916-023-02780-3.

## Background

Gastrointestinal infections, pneumonia, and urinary tract infections are common causes of hospital admission and important causes of death [1]. Identifying modifiable risk factors for those infections is essential since the disease burden is projected to increase due to antibiotic resistance, an aging population, and emerging pathogens [2]. Multiple micronutrients have been established to have vital roles in the immune system and are important components for the proliferation and maturation of immune cells, cytokine release, and enzymes involved in immune cell activity for antioxidant host defense [3]. Deficiency can significantly impair host immunity, increasing susceptibility to infections [3].

Previous observational studies and randomized controlled trials have found that certain micronutrients reduce the risk of specific infections [3]. However, the results are conflicting, possibly due to factors such as high variability between studies and the use of different outcomes. It can be difficult to conduct randomized controlled trials due to logistical issues and costs, and not many adequately powered trials have evaluated the effect of micronutrients and infections. Also, it can be challenging to quantify the causal effects from traditional observational studies due to residual confounding and reverse causation [4].

Mendelian randomization (MR) provides an alternative method to determine evidence of causality. MR uses single-nucleotide polymorphisms (SNPs) identified by genome-wide association studies (GWASs) as genetic instruments to evaluate the effect of an exposure (e.g., blood levels of copper) on the risk of an outcome (e.g., gastrointestinal infection). GWASs have successfully identified several genetic variants involved in the metabolic pathway of several vitamins and minerals [5–15]. Importantly, since these genetic variants are allocated randomly at conception, MR studies are much less susceptible to reverse causation and confounding than traditional observational studies [4].

The aim of this study was to estimate the association between genetically predicted blood levels of micronutrients on the genetically predicted risk of infectious diseases. We identified eight micronutrients of interest that have previously been linked to the risk of infection and for which genetic instruments were available—copper, iron, selenium, zinc, beta-carotene, vitamin B12, vitamin C, and vitamin D—and evaluated the risk of the following three infections: gastrointestinal infections, pneumonia, and urinary tract infections.

## Methods

### Study design

**Fig. 1:**
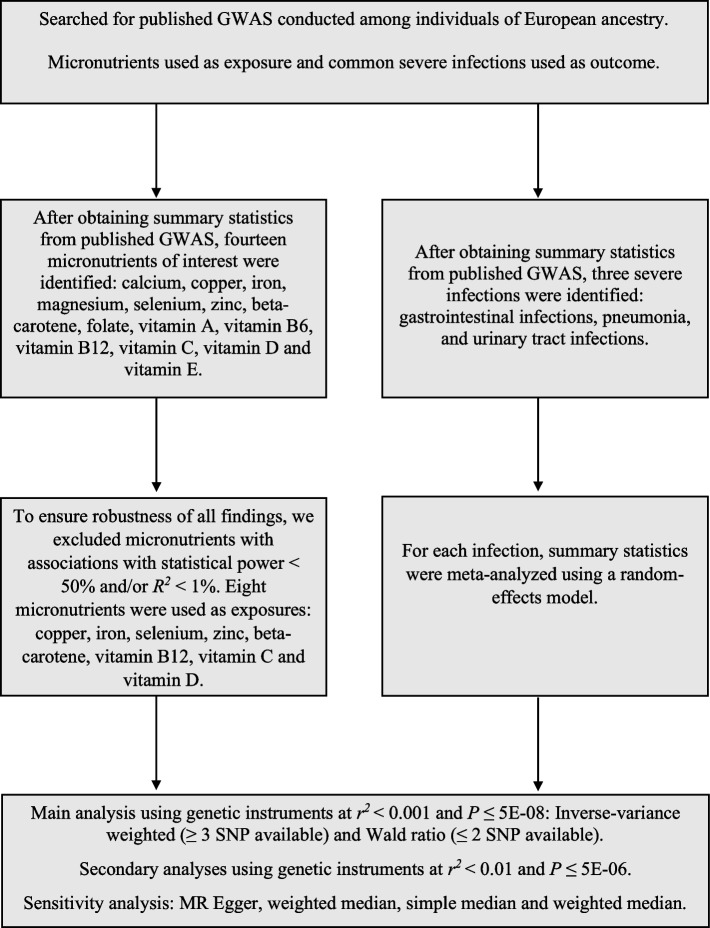
A schematic summary of the study design

This study is reported according to the STROBE-MR (Additional file 1: Table S1) [16]. A schematic summary of the study design is given in Fig. 1. Briefly, we conducted a two-sample MR study using data from publicly available summary statistics from fourteen GWASs: eight for the exposures and six for the outcomes. Both exposure and outcome cohorts were restricted to subjects of European ancestry to reduce bias from population stratification [17]. All data used in this work are publicly available from studies with relevant participant consent and ethical approval, and ethical approval from an institutional review board was therefore not necessary for the present study.

### Data on the genetically predicted levels of circulating micronutrients

We searched for published GWASs evaluating individuals of European ancestry on the GWAS Catalog and PubMed (the last search was performed in May 2022). We did not find any GWAS conducted for vitamins B1, B2, B3, B5, B7, sulfur, iodine, chloride, and fluoride. The GWASs conducted for vitamin K, potassium, sodium, cobalt, chromium, and molybdenum were excluded because of no significant genome-wide results [8, 18, 19]. In total, fourteen micronutrients of potential interest were identified: calcium [5], copper [6], iron [7], magnesium [8], selenium [6], zinc [6], beta-carotene [9], folate [10], vitamin A [11], vitamin B6 [12], vitamin B12 [10], vitamin C [13], vitamin D [14], and vitamin E [15] (Additional file 2: Additional Text) [5–15, 20, 21]. For copper, we also identified a more recent and larger GWAS by Jäger et al. [20], but given that this study reported *Z*-scores and not beta-coefficients, we used the study by Evans et al. [6] in order to improve interpretability. However, the genetic instruments from the GWAS by Jäger et al. [20] were used in secondary analyses. Vitamin A and vitamin E were excluded because those GWASs were adjusted for body mass index (BMI) [22] which might introduce collider bias if the genetic instruments of the exposure of interest also have an effect on BMI [23].

For the main MR analysis, we included independent SNPs (*r*^2^ < 0.001 within 10,000-kb windows), strongly associated (*P* ≤ 5E−08) with the blood level of each micronutrient.

### Data on the genetically predicted risk of infectious diseases

Based on the disease incidence and availability of published summary statistics, we evaluated the risk of the following three infections: gastrointestinal infections, pneumonia, and urinary tract infections. We used publicly available summary statistics from two independent cohorts of European ancestry: UK Biobank (UKBiobank HRC-imputed) [24] and FinnGen Release 6 [25, 26] (Table 1). The GWAS conducted using the UKBiobank HRC-imputed data was performed using SAIGE (a generalized mixed model association test that uses the saddlepoint approximation to account for case-control imbalance), adjusted for genetic relatedness, sex, birth year, and the first four principal components [24]. The GWASs conducted on the FinnGen dataset were analyzed using SAIGE and were adjusted for sex, age, first ten principal components, and genotyping batch [25].

**Table 1:** Source of outcome genome-wide association study summary data

	Gastrointestinal infections			Pneumonia			Urinary tract infections		
	Cases	Controls	Phenocode/name	Cases	Controls	Phenocode/name	Cases	Controls	Phenocode/name
**UK Biobank**	8991	399,970	008 Intestinal infection	6710	398,538	480.1 Bacterial pneumonia	12,491	379,936	591 Urinary tract infections
**FinnGen R6**	25,968	234,437	AB1 Intestinal infections	9878	223,587	J10 Pneumobact	19,479	231,480	N14 Urethraoth
**Meta-analyses**	34,959	634,407	–	16,588	622,125	–	31,970	611,416	–

In both the UK Biobank and FinnGen, cases and controls were defined based on International Classification of Diseases codes (10th revision) from hospital records (Additional file 2: Tables S2-S4) [27–32]. For each infectious disease, summary statistics were meta-analyzed using a random-effects model in METAL (version 2011-03-25) [33] (Additional file 3: Table S5). The meta-analyses included genomic control to account for residual population stratification [33]. Additionally, we conducted Cochran’s *Q* statistical test, included in METAL [33], to assess the heterogeneity between the two cohorts for the genetic instruments used for the outcomes.

### Statistical power

The strength of each genetic instrument was estimated using the *F* statistic: *F* = *R*^2^(*N* − 2)/(1 − *R*^*2*^), where *R*^2^ equals the proportion of variance explained by the genetic instrument and *N* is the effective sample size of the GWAS for the SNP-micronutrient association [34]. The *R*^2^ value was calculated using the formula 2 × MAF(1 − MAF)beta^2^, where beta represents the effect estimate of the genetic variant in the exposure, measured in standard deviation (SD) units, and MAF represents the minor allele frequency [35] (Table 2). The effect allele frequency was not available for the genetic instruments used for copper, selenium, and zinc, published by Evans et al. [6]. However, for the main analysis, none of the copper or selenium-associated SNPs were palindromic (A/T or G/C alleles), so it was clear which allele was the effect allele. For zinc, we removed one genetic instrument, rs10931753, due to being palindromic. The effect allele frequencies from the meta-analysis of FinnGen and UK Biobank were used to estimate the *F* statistics and *R*^2^ for copper, selenium, and zinc. Although this may result in incorrect calculations of *R*^2^ (and thus *F* statistic and statistical power), our results aligned well with the reported *R*^2^ from Evans et al. [6], with an *R*^2^ of 5% for copper. We reported a lower *R*^2^ value for selenium (*R*^2^ of 2.4%), but we only used 1 SNP, while Evans et al. [6] reported a total *R*^2^ for 2 SNPs (*R*^2^ of 4%) and a lower *R*^2^ value for zinc (*R*^2^ of 4.25%), where we used 2 SNPs, while Evans et al. [6] reported a total *R*^2^ for 3 SNPs (*R*^2^ of 8%). Additionally, for the secondary analysis of copper, we observed an unlikely *R*^2^ which was explained by one extremely outlying SNP: rs12582659 (Additional file 2: Fig. S1). After removing this SNP, we found an *R*^2^ of 7.10%.

**Table 2:** Source of exposure genome-wide association study summary data

Exposure	Main analysis^**a**^		Seconary analysis^**b**^			Population ancestry	Reference
	Number of SNPs	% of variance explained		Number of SNPs	% of variance explained		
Ca^c^	2	0.15		3	0.18	European	[5]
Cu	2	4.36	QIMR	6	7.10^d^	European	[6, 20]
			Jäger et al.	7	6.55	European	
Fe	13	3.01		96	5.37	European	[7]
Mg^c^	6	0.01		8	0.01	European	[8]
Se	1	2.40	QIMR	6	10.2	European	. [6]
			ALSPAC	7	8.06	European	
Zn	2	4.25		7	8.66	European	[6]
Beta-carotene	1	4.59		–	–	European	[9]
Folate^c^	2	0.58		3	0.67	European	[10]
Vitamin B6^c^	1	0.67		–	–	European	[12]
Vitamin B12	10	4.36		11	4.43	European	[10]
Vitamin C	11	1.90		20	2.28	European	[13]
Vitamin D	59	1.98		75	2.39	European	[14, 15]

Missing data is denoted by “–”

*Abbreviations*: *Ca* calcium, *Cu* copper, *Fe* iron, *Mg* magnesium, *Se* selenium, *Zn* zinc

^a^For the main analyses, only independent (*r*^2^ < 0.001 within 10,000-kb windows) and strongly associated (*P* ≤ 5E−08) variants were used

^b^For the secondary analyses, only variants (*r*^2^ < 0.01 within 10,000-kb windows) suggestively associated (*P* ≤ 5E−06) variants were used

^c^Ca, Mg, and folate were excluded due to the variance explained < 1%

^d^We observed extreme values for *R*^2^, which was explained by one extremely outlying SNP; rs12582659 had an *R*^2^ of 17.80%

Power calculations were done using http://cnsgenomics.com/shiny/mRnd/ [36]. The statistical power was calculated to capture an odds ratio (OR) of 0.90 or 1.10 per SD change in the circulating micronutrient concentration, given the sample size used for the meta-analyses at a type 1 error of 5% (Additional file 2: Table S6). In addition to the main MR analyses, we performed secondary analyses using more liberal criteria for including genetic variants to enhance statistical power; *r*^2^ < 0.01 and *P* ≤ 5E−06. For our study, we only considered micronutrients with an *R*^2^ > 1% and/or statistical power > 50% for at least one of the infectious disease outcomes, thereby excluding calcium, magnesium, folate, and vitamin B6 (Table 2 and Additional file 2: Tables S6-S7). For the remaining micronutrients, we excluded SNPs with an *F* statistic < 10 [4] to reduce the risk of weak instrument bias [34]. None of the included genetic instruments was shared by any of the considered micronutrients (Additional file 2: Table S7).

### MR analysis

We calculated the Wald ratio for each SNP, defined as the SNP-outcome association divided by the SNP-exposure association [37]. When multiple SNPs were available for a micronutrient, we summarized the effect calculated by the Wald ratio using an inverse-variance weighted (IVW) analysis [38]. All reported associations correspond to an OR for the outcome per SD increase in the genetically predicted circulating concentrations of the micronutrient. The MR analyses were performed separately for the outcome GWASs from UK Biobank, FinnGen, and meta-analysis of the two cohorts. If not otherwise specified, the meta-analysis was used as the outcome study. *P* < 0.05 was considered nominally significant, whereas the level for statistical significance corrected for multiple testing (8 exposures × 3 outcomes = 24 tests) was set at *P* = 0.05/24 = 2.08E−03.

### Sensitivity analyses

For an instrumental variable to be valid, three key assumptions must be met: the instrument must be robustly associated with the exposure, it cannot affect a confounder of the exposure-outcome association, and it must only affect the outcome through the risk factor [4]. Horizontal pleiotropy—that the SNP has multiple effects—can violate those assumptions. MR-Egger, weighted median, simple mode, and weighted mode are some of the most common sensitivity analyses to account for horizontal pleiotropy [17]. These analyses were only conducted when the number of genetic instruments was ≥ 3. The MR-Egger method allows some SNPs to affect the outcome through a pathway other than through the exposure. If the intercept term differs from zero, this indicates that not all the included instruments are valid, and the standard estimates (i.e., IVW) may be biased [39]. The weighted median method provides a valid MR estimate when up to 50% of the included instruments are invalid. This method calculates the weighted median estimate by ordering the genetic variants according to the magnitude of their estimates [40]. The mode-based methods (simple mode and weighted mode) assume that the most common causal effect is consistent with the true causal effect, allowing some instruments to be invalid without biasing the estimated causal effect [41].

To evaluate if the differences in the individual effect sizes among the genetic instruments may be related to pleiotropic effects rather than chance, we conducted Cochran’s *Q* statistical test [42]. This test was only conducted when two or more variants were available, and a *P* < 0.05 was considered significant in the test for heterogeneity.

We further evaluated whether the genetic instruments were associated with other phenotypes using PhenoScanner V2 [43], available at http://www.phenoscanner.medschl.cam.ac.uk/ (accessed 30 October 2022). Additionally, we performed leave-one-out analyses for micronutrients containing > 2 SNPs. This was performed to examine the robustness of the IVW estimates and if any specific SNP drove the association (which could be due to pleiotropy) [4].

Multivariable MR was used to evaluate whether any of the identified phenotypes on PhenoScanner had introduced bias due to pleiotropy [17]. The genetic variants for the potentially pleiotropic phenotypes were collected from IEU OpenGWAS [44]. Based on the identified potentially pleiotropic pathways, we conducted one multivariable MR analysis of copper on the risk of gastrointestinal infections where we included erythrocyte count (GWAS identifier: ukb-d-30250_irnt) and hemoglobin concentration (GWAS identifier: ebi-a-GCST004615) in the analysis.

### Secondary analyses using less stringent criteria for the selection of genetic instruments

In secondary analyses, we included variants at a more liberal threshold of *r*^2^ < 0.01 and *P* ≤ 5E−06. While this could increase statistical power, it also may increase the risk of violating the MR assumptions and introduce weak instrument bias. Therefore, we included MR-RAPS, an MR method for correcting for bias introduced by weak instruments, using robust adjusted profile scores [45]. For copper, we carried out the secondary analyses both using instruments from Evans et al. [6] (as in the main analysis) and using instruments from Jäger et al. [20].

### Post hoc analyses

Finally, to validate the association between copper and gastrointestinal infections (see the “Results” section), we conducted the following two post hoc analyses: first, we retrieved the results from an additional European GWAS on gastrointestinal infections conducted by Nudel et al. [46] and conducted MR analyses on this independent cohort. Only one of the two copper SNPs, rs2769264, was available from this study, and no reliable proxy for the other copper SNP was available (defined by *r*^2^ > 0.9; using European ancestry in the LDproxy tool from National Cancer Institute LDlink [47]).

Second, to assess the possibility of reverse causation, we conducted MR analyses of the association between the genetically predicted risk of gastrointestinal infection on the genetically predicted blood levels of copper. We used the meta-analysis results on gastrointestinal infection as exposure in this analysis, including SNPs suggestively associated with gastrointestinal infection (*r*^2^ < 0.01 within 10,000-kb windows, *P* ≤ 5E−06). For the outcome, we retrieved the copper association summary-level statistics from Evans et al. [6].

### Statistical analysis

All MR analyses were conducted using the TwoSampleMR package (version 0.5.6) [42] in R (version 4.0.3). METAL (version 2011-03-25) [33] was used to perform the meta-analyses of the outcomes.

## Results

### Main analyses

**Fig. 2:**
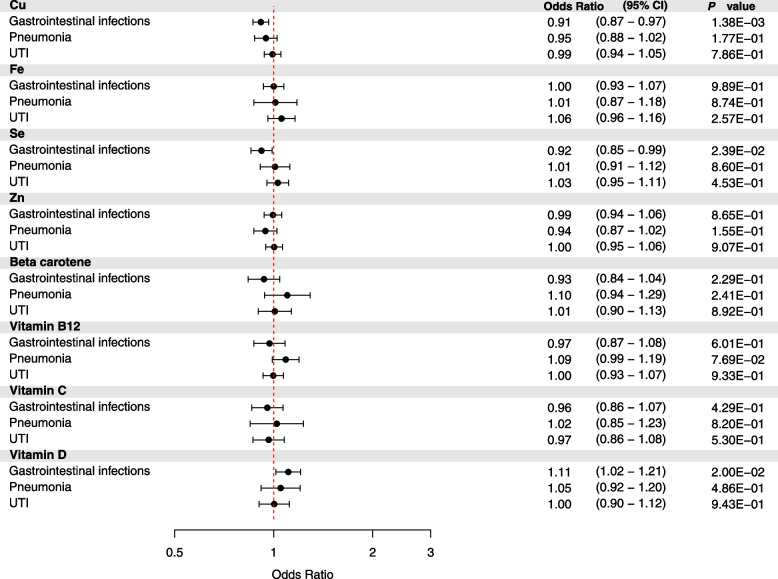
Mendelian randomization analyses of circulating levels of micronutrients on the risk of gastrointestinal infections, pneumonia, and urinary tract infections. *Legend*: Forest plot of inverse-variance weighted Mendelian randomization analyses. The *x*-axis represents the results expressed as per standard deviation increase in genetically proxied levels of the exposure. *Abbreviations*: Cu, copper; Fe, iron; Se, selenium; UTI, urinary tract infection; Zn, zinc

After correction for multiple testing, the only statistically significant micronutrient-infection association was that of genetically predicted blood levels of copper and risk of gastrointestinal infections (Fig. 2). One SD increase in genetically predicted blood levels of copper was associated with an OR of 0.91 (95% confidence interval [CI] 0.87 to 0.97, *P* = 1.38E−03), 0.89 (95% CI 0.80 to 0.98, *P* = 1.67E−02), and 0.93 (95% CI 0.87 to 0.99, *P* = 1.98E−02), in the meta-analysis, UK Biobank, and FinnGen, respectively (Fig. 2 and Additional file 2: Table S8). A nominally significant association was observed for both selenium and vitamin D on the risk of gastrointestinal infections, with an OR of 0.92 (95% CI 0.85 to 0.99, *P* = 2.39E−02) and OR of 1.11 (95% CI 1.02 to 1.21, *P* = 2.00E−02), respectively (Fig. 2 and Additional file 2: Table S8).

We observed little evidence that the circulating concentrations of iron, zinc, beta-carotene, vitamin B12, and vitamin C were associated with the risk of any of the evaluated infections (Fig. 2 and Additional file 2: Tables S8-S10).

### Sensitivity and secondary analyses

The MR-Egger, weighted median, and mode-based sensitivity analyses supported the findings from the IVW analyses. As only two instruments were used for copper, MR-Egger regression, weighted median, simple mode, and weighted mode were not carried out in the main analysis. No heterogeneity was observed in the main MR analyses for copper and vitamin D on the risk of gastrointestinal infections (Cochran’s *Q* test *P* = 5.99E−01 and Cochran’s *Q* test *P* = 5.56E−01, respectively; Additional file 2: Table S8). Heterogeneity was observed for vitamin B12 and gastrointestinal infections (Cochran’s *Q* test *P* = 2.19E−04): iron (Cochran’s *Q* test *P* = 1.04E−02) and vitamin C (Cochran’s *Q* test *P* = 3.70E−02) for pneumonia and vitamin D (Cochran’s *Q* test *P* = 2.27E−02) for urinary tract infection. For the other micronutrients, no heterogeneity was observed (Additional file 2: Tables S8-S10).

For the secondary analyses, we generally observed comparable effects as in the main analyses (Additional file 2: Table S11). However, for copper, using 6 SNPs from Evans et al. [6] yielded an IVW estimate of OR 1.01 (95% CI 0.96 to 1.06, *P* = 7.45E−01). There was considerable heterogeneity in this estimate (Cochran’s *Q* test *P* = 4.68E−03), which was explained by one extremely outlying SNP: rs12582659 (Additional file 2: Fig. S1). Excluding this SNP from the analysis yielded comparable results to the main analysis (OR 0.97, 95% CI 0.90 to 1.05, *P* = 4.35E−01; Additional file 2: Table S12). However, we still found evidence of heterogeneity (Cochran’s *Q* test *P* = 1.47E−02), and the results should be interpreted with caution. Using genetic instruments from Jäger et al. [20] supported the main analysis (OR 0.95, 95% CI 0.91 to 1.00, *P* = 3.07E−02). In both secondary analyses using data from Evans et al. [6] and Jäger et al. [20], we observed the presence of potential pleiotropy, e.g., MR-Egger OR 1.01 (95% CI 0.94 to 1.09, *P* = 7.18E−01) and OR 1.07 (95% CI 0.93 to 1.23, *P* = 3.99E−01); it is unclear whether this pleiotropy reflects the presence of pleiotropy in the main analysis.

Using PhenoScanner, we found that several of the genetic instruments used for the micronutrients have previously been reported to be associated with numerous traits and diseases (Additional file 3: Tables S13-S20). Of note, rs1175550 used for copper was strongly associated with reticulocyte count and hemoglobin concentration. We therefore performed a multivariable MR analysis between copper, reticulocyte count, and hemoglobin concentration and the risk of gastrointestinal infection. We observed a similar effect as in the main analysis with an OR of 0.96 (95% CI 0.93 to 0.99, *P* = 1.64E−02). The genetic instruments for vitamin D were associated with several traits related to smoking, BMI, and alcohol. In the leave-one-out analysis, the observed association of vitamin D with gastrointestinal infection did not change meaningfully, indicating that no specific SNP drove the result nor that the observed association was due to pleiotropy (Additional file 2: Table S21).

### Post hoc analyses

In the post hoc analysis of copper and gastrointestinal infections, we observed a similar effect as in the main analysis when using another GWAS on gastrointestinal infections [46]: OR 0.94 (95% CI 0.81 to 1.09, *P* = 3.82E−01). The CI was wide because only one of the two SNPs was available. Meta-analyzing the results from using Nudel et al. [46] with the main analysis yielded an OR of 0.92 (95% CI 0.87 to 0.97, *P* = 1.14E−03).

Finally, we conducted two-sample MR analyses using gastrointestinal infections as the exposure and blood levels of copper as the outcome. We found that gastrointestinal infections did not affect circulating copper levels (beta = − 0.35, 95% CI − 1.35 to 0.71, *P* = 5.40E−01) using two genetic instruments from Evans et al. [6], and no heterogeneity was observed (Cochran’s *Q* test *P* = 5.50E−01).

## Discussion

In this MR study of eight micronutrients and the risk of three infectious diseases, we found genetically predicted blood levels of copper to be robustly associated with the genetically predicted risk of gastrointestinal infections. We did not find a clear association between the other micronutrients and infections.

Copper plays an essential role in innate and adaptive immunity: it regulates the function of T helper cells, B cells, neutrophils, natural killer cells, and macrophages; it accumulates at sites of inflammation, including the gastrointestinal and respiratory tract and in blood and urine, and is vital for interleukin 2 production and response [3, 48]. Blood levels of copper have not previously been robustly linked to the risk of gastrointestinal infections in humans. A small randomized controlled trial (RCT) found that supplementation with high doses of copper, zinc, and selenium significantly reduced the risk of infections among hospitalized patients with severe burns [49]. Another trial found that copper supplementation increased the interleukin 2 production by blood cells in healthy individuals with low to normal copper levels, which is crucial for T helper cell proliferation and natural killer cell cytotoxicity [50]. In addition, a previous study showed that cell cultures pretreated with added Cu boosted macrophage antibacterial activity and enhanced intracellular killing of *Escherichia coli* [51]. These results align with our finding that high levels of copper have a protective effect against infectious diseases and that higher blood levels of copper might lead to increased immune response.

Regarding vitamin D, a previous MR study found that lower plasma levels of this micronutrient were associated with an increased risk of pneumonia [52], which was not supported in our study and also not supported by a systematic review of trials of vitamin D supplementation [53]. The same MR study found no evidence of an association between vitamin D and the risk of urinary tract infections or gastroenteritis [52]. While we also found no association between vitamin D and urinary tract infections, we did observe a nominally significant positive association between vitamin D and gastrointestinal infection. However, this finding may be a chance finding due to multiple testing, and it did not pass our stringent threshold for statistical significance.

Interestingly, we found no associations between genetically predicted circulating iron, zinc, beta-carotene, vitamin B12, and vitamin C and the risk of gastrointestinal infections, pneumonia, or urinary tract infection. Systematic reviews of RCTs have found limited evidence of micronutrient supplementation on the risk of infections but have also underscored the paucity of studies [54–57]. Among those reviews, one reported no difference in the incidence of diarrhea and lower respiratory tract infection in infants with zinc supplementation [54]. Another review found uncertain and limited evidence for vitamin C supplementation in preventing pneumonia [55]. Two reviews found no clear evidence that emerged in favor of selenium supplementation for developing infections [56] and the incidence of new infections [57] among critically ill patients. This may indicate that several of these micronutrients are not important risk factors for the infections considered. Finally, high levels of serum iron have in previous MR studies been associated with skin and soft tissue infections and sepsis, but we did not find any evidence of an association for the infections that we considered [58, 59]. This discrepancy may be due to organ-specific effects of iron (e.g., iron levels were also associated with damages to skin-related structures) and that the infectious diseases are not comparable (e.g., sepsis is an inflammatory syndrome in response to severe infection) [59, 60].

Our study has several strengths and limitations. By applying an MR design, we reduced the risk of confounding, which often affects observational studies. Additionally, we considerably reduced random error and increased statistical power by combining summary data from multiple cohorts [35]. However, despite the large sample sizes, several of the genetic instruments used for exposures and the outcomes, to a varying degree, suffered from low statistical power and imperfect phenotype definitions, which may contribute to the null findings of the majority of associations explored. Larger GWASs on micronutrients and infections, with more precise phenotype definitions, would be beneficial. Also, summarized data does not allow for stratification by factors such as sex, age, diet, micronutrient supplement use, or co-morbidities. Due to the use of summary-level data, we could not identify individuals with a combination of two or more infections, which might lead to bias. The quality control, genotyping, and imputation were performed using different criteria and programs for the two cohorts. Additionally, different phenotype definitions were used in the two cohorts, which may introduce heterogeneity between the association estimates. However, we observed minimal heterogeneity between the two cohorts in the meta-analysis.

The genetic instruments used as exposure for each micronutrient have widely been used to evaluate the association with other complex diseases or phenotypes, which supports their use in this study [61–63]. Throughout, we tried to use data on our exposures and outcomes from separate GWASs to reduce the risk of confounding bias due to overlapping samples [64], but this was not possible for vitamin D (since the other published GWASs for vitamin D adjusted for BMI) [21, 65]. To reduce the risk of population stratification, we only evaluated participants of European ancestry. However, this affects our findings’ external validity to other ancestry groups. Our findings were supported by conducting a range of sensitivity analyses, including evaluating the presence of pleiotropy, and by evaluating two distinct biobanks for each outcome (i.e., UK Biobank and FinnGen). While only two instruments were available for the main MR analysis of copper, the more liberal threshold for SNP inclusion in the secondary analyses allowed for more genetic instruments to be included; these analyses were generally consistent with the main analysis. For copper and risk of gastrointestinal infections, we conducted an extended set of sensitivity analyses to evaluate the robustness of our findings, including following up our results in an additional GWAS of gastrointestinal infections, conducting multivariable MR to account for potentially pleiotropic pathways, and conducting bi-directional MR: These analyses all supported our main finding.

## Conclusions

In conclusion, our findings support that copper may play a role in the susceptibility to gastrointestinal infections. More research is needed to evaluate whether this finding replicates in other settings and to learn more about the potential underlying mechanisms.

## Supplementary Information

**Additional file 1: Table S1.** STROBE MR.

**Additional file 2: **Additional Text – Exposure GWAS cohorts. **Fig. S1.** Scatter plot of secondary MR analysis of copper as risk factors on the risk of gastrointestinal infections. **Table S2.** ICD-10 codes for gastrointestinal infections in UK Biobank and FinnGen R6. **Table S3.** ICD-10 codes for pneumonia in UK Biobank, and FinnGen. **Table S4.** ICD-10 codes for urinary tract infection UK Biobank, and FinnGen. **Table S6.** Power calculations. **Table S7.** Genetic variants used as exposure for Mendelian randomization analyses. **Table S8.** Main mendelian randomization analyses of micronutrients as risk factors on the risk of gastrointestinal infections. **Table S9.** Main mendelian randomization analyses of micronutrients as risk factors on the risk of pneumonia. **Table S10.** Main mendelian randomization analyses of micronutrients as risk factors on the risk of urinary tract infections. **Table S11.** Secondary mendelian randomization analyses of micronutrients as risk factors on the risk of gastrointestinal infections, pneumonia and urinary tract infections suggestive-significant genetic instruments. **Table S12.** Secondary mendelian randomization analyses of copper as risk factors on the risk of gastrointestinal infections, where rs12582659 was removed. **Table S21.** IVW MR regression results for the leave one SNP out analysis in the Mendelian randomization analyses of micronutrients.

**Additional file 3: Table S5.** Results from the meta-analysis for the genetic instruments used as the outcome in the Mendelian randomization analyses. **Table S13.** Phenome-wide association analysis of genetic instruments for copper. **Table S14.** Phenome-wide association analysis of genetic instruments for iron. **Table S15.** Phenome-wide association analysis of genetic instruments for selenium. **Table S16.** Phenome-wide association analysis of genetic instruments for zinc. **Table S17.** Phenome-wide association analysis of genetic instruments for beta carotene. **Table S18.** Phenome-wide association analysis of genetic instruments for vitamin B12. **Table S19.** Phenome-wide association analysis of genetic instruments for vitamin C. **Table S20.** Phenome-wide association analysis of genetic instruments for vitamin D.

## Data Availability

Data described in the manuscript are provided within the article. Genetic instrumental variables and data sources are presented in the additional files. The UKBiobank HRC-imputed can be obtained via https://pheweb.org/UKB-SAIGE/. The summary-level data for FinnGen can be obtained via https://www.finngen.fi/en/access_results. We used the following web-based resources: Phenoscanner (http://www.phenoscanner.medschl.cam.ac.uk/), LDlink (https://analysistools.cancer.gov/LDlink/?tab=ldproxy), and IEU OpenGWAS (https://gwas.mrcieu.ac.uk/).

## Abbreviations

BMI: Body mass index
CI: Confidence interval
GWAS: Genome-wide association study
ICD: International Classification of Diseases Code
IVW: Inverse-variance weighted
MAF: Minor allele frequency
MR: Mendelian randomization
OR: Odds ratio
RCT: Randomized controlled trial
SD: Standard deviation
SNP: Single-nucleotide polymorphism
