# Supplementary material for: Circulating levels of micronutrients and risk of infections: a Mendelian randomization study
Source: BMC Med. 2023 Mar 8;21:84. doi: 10.1186/s12916-023-02780-3 (PMC9993583; doi:10.1186/s12916-023-02780-3)
Supplement: Supplementary file 1 — Additional file 1: Table S1. STROBE MR. [file 12916_2023_2780_MOESM1_ESM.docx]

**Additional File 1.**

**Table S1.** STROBE-MR checklist of recommended items to address in reports of Mendelian randomization studies.

| Item No. | Section | Checklist item | Page No. | Relevant text from manuscript |
| --- | --- | --- | --- | --- |
| 1 | TITLE and ABSTRACT | Indicate Mendelian randomization (MR) as the study’s design in the title and/or the abstract if that is a main purpose of the study | 1 | Circulating levels of micronutrients and risk of infections: A Mendelian randomization study |
|  | INTRODUCTION |  |  |  |
| 2 | Background | Explain the scientific background and rationale for the reported study. What is the exposure? Is a potential causal relationship between exposure and outcome plausible? Justify why MR is a helpful method to address the study question | 4-5 | Micronutrients play essential roles in supporting the immune system, and deficiencies can therefore lead to increased susceptibility to infections.  Previous observational studies and randomized controlled trials have found that certain micronutrients reduce the risk of specific infections, such as increased intake of zinc, vitamin D, and vitamin E on the risk of pneumonia. |
| 3 | Objectives | State specific objectives clearly, including pre-specified causal hypotheses (if any). State that MR is a method that, under specific assumptions, intends to estimate causal effects | 5 | We performed a Mendelian randomization analysis to complement observational and RCT findings. The aim of this study was to estimate the association between genetically-predicted blood levels of micronutrients on the genetically-predicted risk of infectious diseases. We evaluated the risk of the following three predominantly bacterial infections: gastrointestinal infections, pneumonia, and urinary tract infections. Similarly, we identified eight micronutrients of interest: copper, iron, selenium, zinc, beta-carotene, vitamin B12, vitamin C, and vitamin D. |
|  | METHODS |  |  |  |
| 4 | Study design and data sources | Present key elements of the study design early in the article. Consider including a table listing sources of data for all phases of the study. For each data source contributing to the analysis, describe the following: |  |  |
|  | a) | Setting: Describe the study design and the underlying population, if possible. Describe the setting, locations, and relevant dates, including periods of recruitment, exposure, follow-up, and data collection, when available. | 6-7  6-7  7 | See Figure 1 for a schematic summary of the study design.  Exposure: Additional File 2 Additional text, Table 2 and Additional File 2 Table S6.  Outcome: We used publicly available summary statistics from two independent cohorts of European ancestry: UK Biobank (UKBiobank HRC-imputed) and FinnGen (R6 release) (Table 1). Cases and controls were defined based on ICD10-codes (Additional File 2 Table S2-S4). |
|  | b) | Participants: Give the eligibility criteria, and the sources and methods of selection of participants. Report the sample size, and whether any power or sample size calculations were carried out prior to the main analysis | 8-9 | Power calculations were calculated (Additional File 2 Table S6).  Exposure: Additional File 2 Additional text, Table 2, and, Table S7.  Outcome: Table 1 and Additional File 2 Table S2-S4. |
|  | c) | Describe measurement, quality control and selection of genetic variants | 6-9 | To limit the risk of weak instrument bias micronutrients with statistical power < 50% and R2 < 1% were excluded. For the remaining eight micronutrients, we excluded SNPs with an F statistic < 10 (Table 2, Additional File 2 Tables S6-S7). Only independent SNPs (r2 < 0.001 within 10,000 kb windows), strongly associated (*P* ≤ 5E-08) with the blood level of each micronutrient, were used in the main analyses (Additional File 2 Table S7). |
|  | d) | For each exposure, outcome, and other relevant variables, describe methods of assessment and diagnostic criteria for diseases | 6-9  7 | Exposure: Additional File 2 Additional text, Table 2 and Additional File 2 Table S7.  Outcome: Cases and controls were defined based on ICD10-codes (Table 1 and Additional File 2 Tables S2-S4). |
|  | e) | Provide details of ethics committee approval and participant informed consent, if relevant | 6 | All participants provided informed consent in all the corresponding original studies. All data used in this work are publicly available from studies with relevant participant consent and ethical approval. Ethical approval from an institutional review board was not necessary for the present study as only publicly available summary level data was used. |
| 5 | Assumptions | Explicitly state the three core IV assumptions for the main analysis (relevance, independence and exclusion restriction) as well assumptions for any additional or sensitivity analysis | 9  9-10 | Three key assumptions explained  Sensitivity analyses explained |
| 6 | Statistical methods: main analysis | Describe statistical methods and statistics used |  |  |
|  | a) | Describe how quantitative variables were handled in the analyses (i.e., scale, units, model) | NA |  |
|  | b) | Describe how genetic variants were handled in the analyses and, if applicable, how their weights were selected | NA |  |
|  | c) | Describe the MR estimator (e.g. two-stage least squares, Wald ratio) and related statistics. Detail the included covariates and, in case of two-sample MR, whether the same covariate set was used for adjustment in the two samples | 9  6-7 | IVW  Both exposure and outcome GWASs were adjusted for sex, age, and PCs.  Additional File 2 Additional text for exposure. |
|  | d) | Explain how missing data were addressed | NA |  |
|  | e) | If applicable, indicate how multiple testing was addressed | 9 | P values < 0.05 were considered nominally significant, whereas high-confidence findings were those that survived a stringent Bonferroni correction to account for multiple testing (8 exposure x 3 outcomes = 24 tests), setting the following threshold for statistical significance: *P* = 0.05/24 = 2.08E-03. |
| 7 | Assessment of assumptions | Describe any methods or prior knowledge used to assess the assumptions or justify their validity | 7 | Only independent SNPs (r2 < 0.001 within 10,000 kb windows), strongly associated (*P* ≤ 5E-08) with the blood level of each micronutrient, were used (Table 2 and Additional File 2 Table S7). |
| 8 | Sensitivity analyses and additional analyses | Describe any sensitivity analyses or additional analyses performed (e.g. comparison of effect estimates from different approaches, independent replication, bias analytic techniques, validation of instruments, simulations) | 9-11  11  7 and 10  10  10 | We conducted sensitivity analyses to account for pleiotropy for genetic instruments with ≥ 3 variants: MR Egger, weighted median, simple mode, and weighted mode (Additional File 2 Tables S8-S10).  We performed secondary analyses, including variants at a more liberal, suggestive-significant threshold at r2 < 0.01 and *P* ≤ 5E-06. This allowed us to enhance the statistical power of the analyses (Additional File 2 Tables S11-S12).  We conducted the Cochran's Q statistical test to assess heterogeneity (Additional File 3 Table S5 and Additional File 2 Tables S11-S12).  For exposures that were significantly associated with an outcome, we looked up each exposure SNP in the Phenoscanner V2 (accessed 30th October 2022), to evaluate whether the association could be due to pleiotropy. We found that copper was associated with reticulocyte count. We, therefore, performed multivariable analysis for copper and gastrointestinal infection, adjusting for reticulocyte count (Additional File 3 Tables S13-S20).  We performed leave-one-out analyses to examine the robustness of the IVW estimates and whether any specific SNP drove the association (Additional File 2 Table S21) |
| 9 | Software and pre-registration |  |  |  |
|  | a) | Name statistical software and package(s), including version and settings used | 12  7  8-9  10 | The TwoSampleMR R package (version 0.5.6) in R (version 4.0.3) was used to conduct all MR analyses.  METAL (version 2011-03-25) was used to perform the meta-analyses for the outcomes.  Power calculations were calculated post-hoc using an online tool available at <http://cnsgenomics.com/shiny/mRnd/>  Phenoscanner V2, available at <http://www.phenoscanner.medschl.cam.ac.uk/>, was used to evaluate whether the association could be due to pleiotropy. |
|  | b) | State whether the study protocol and details were pre-registered (as well as when and where) |  | No. |
|  | RESULTS |  |  |  |
| 10 | Descriptive data |  |  |  |
|  | a) | Report the numbers of individuals at each stage of included studies and reasons for exclusion. Consider use of a flow diagram |  | Exposure: Additional File 2 Additional text, Additional File 2 Table S7.  Outcome: Table 1. |
|  | b) | Report summary statistics for phenotypic exposure(s), outcome(s), and other relevant variables (e.g. means, SDs, proportions) |  | Exposure: Additional File 2 Additional text and Additional File 2 Table S7.  Outcomes: Table 1 and Additional File 2 Table S5. |
|  | c) | If the data sources include meta-analyses of previous studies, provide the assessments of heterogeneity across these studies |  | No heterogeneity was observed for the for the three copper SNPs between the two cohorts included in the meta-analysis (I2 = 0% for the two Cu SNPs, Cochran’s Q test *P* = 5.06E-01 for rs2769264 and *P* = 7.85E-01 for rs1175550, see Additional File 3 Table S5) |
|  | d) | For two-sample MR:  i.  Provide justification of the similarity of the genetic variant-exposure associations between the exposure and outcome samples  ii.  Provide information on the number of individuals who overlap between the exposure and outcome studies |  | Applies only to a minimal number of participants. i. We used two different data sources for exposures and outcomes, expect for vitamin D. We calculated the Cochran's Q-test for heterogeneity to assess heterogeneity across the cohorts and found minimal heterogeneity for the included variants for the outcomes (Additional File 3 Table S5 and Additional File 2 Tables S8-S10)  ii. Due to the use of summary-level statistics we were not able to identify individuals who overlap between the exposure and outcome for vitamin D. |
| 11 | Main results |  |  |  |
|  | a) | Report the associations between genetic variant and exposure, and between genetic variant and outcome, preferably on an interpretable scale |  | Figure 2, Additional File 2 Table S8-S10 for main analyses, and Additional File 2 Table S11-S12 for secondary analyses using r2 < 0.01 within 10,000 kb windows and *P* ≤ 5E-06. |
|  | b) | Report MR estimates of the relationship between exposure and outcome, and the measures of uncertainty from the MR analysis, on an interpretable scale, such as odds ratio or relative risk per SD difference |  | Figure 2, Additional File 2 Table S8-S10 for main analyses, and Additional File 2 Table S11-S12 for secondary analyses using r2 < 0.01 within 10,000 kb windows and *P* ≤ 5E-06. |
|  | c) | If relevant, consider translating estimates of relative risk into absolute risk for a meaningful time period | NA |  |
|  | d) | Consider plots to visualize results (e.g. forest plot, scatterplot of associations between genetic variants and outcome versus between genetic variants and exposure) |  | Figure 2. |
| 12 | Assessment of assumptions |  |  |  |
|  | a) | Report the assessment of the validity of the assumptions | 13-15 | Methods to assess the robustness of MR findings: MR Egger, weighted median, simple mode, weighted mode, Cochran's Q statistical test, PhenoScanner, leave-one-out analyses and post-hoc analyses. |
|  | b) | Report any additional statistics (e.g., assessments of heterogeneity across genetic variants, such as I2, Q statistic or E-value) | 13-15 | For the MR analyses, heterogeneity was reported for all analyses consisting of ≥ 2 SNPs (Additional File 2 Table S8-S10). We also reported the Cochran’s Q for the meta-analysis results for the respective SNPs included in the analyses (Additional File 3 Table S5). |
| 13 | Sensitivity analyses and additional analyses |  |  |  |
|  | a) | Report any sensitivity analyses to assess the robustness of the main results to violations of the assumptions | 13-15 | Additional File 2 Table S8-S10 for the main analyses. Additional File 2 Table S11-S12 for MR analyse using SNPs with a liberal suggestive-significant threshold at r2 < 0.01 and *P* ≤ 5E-06. |
|  | b) | Report results from other sensitivity analyses or additional analyses | 14-15 | By using the PhenoScanner platform, we found that several of the SNPs were associated with secondary phenotypes (Additional File 3 Tables S13-S20). The two copper SNPs previously been reported to be associated with traits related to reticulocytes and hemoglobin (Additional File 3 Table S13). |
|  | c) | Report any assessment of direction of causal relationship (e.g., bidirectional MR) | 15 | We conducted a bi-directional two-sample MR analysis using gastrointestinal infections as the exposure and blood levels of copper as the outcome. For the outcome, we retrieved the copper-association summary-level statistics from Evans et al. We found that gastrointestinal infections did not affect circulating copper levels. |
|  | d) | When relevant, report and compare with estimates from non-MR analyses | NA |  |
|  | e) | Consider additional plots to visualize results (e.g., leave-one-out analyses) |  | Leave-one-out results are presented in Additional File 2 Table S21. |
|  | DISCUSSION |  |  |  |
| 14 | Key results | Summarize key results with reference to study objectives | 16 | We found genetically predicted blood levels of copper to be robustly associated with genetically-predicted risk of gastrointestinal infections. We did not find a clear association between the other micronutrients and infections. |
| 15 | Limitations | Discuss limitations of the study, taking into account the validity of the IV assumptions, other sources of potential bias, and imprecision. Discuss both direction and magnitude of any potential bias and any efforts to address them | 17-18 |  |
| 16 | Interpretation |  |  |  |
|  | a) | Meaning: Give a cautious overall interpretation of results in the context of their limitations and in comparison with other studies | 16-17 |  |
|  | b) | Mechanism: Discuss underlying biological mechanisms that could drive a potential causal relationship between the investigated exposure and the outcome, and whether the gene-environment equivalence assumption is reasonable. Use causal language carefully, clarifying that IV estimates may provide causal effects only under certain assumptions | 16 | Considering that copper has an important role in the development and maintenance of the immune system, it is biologically plausible that copper might influence the risk of gastrointestinal infections. |
|  | c) | Clinical relevance: Discuss whether the results have clinical or public policy relevance, and to what extent they inform effect sizes of possible interventions | 16-17 |  |
| 17 | Generalizability | Discuss the generalizability of the study results (a) to other populations, (b) across other exposure periods/timings, and (c) across other levels of exposure | 17-18 |  |
|  | OTHER INFORMATION |  |  |  |
| 18 | Funding | Describe sources of funding and the role of funders in the present study and, if applicable, sources of funding for the databases and original study or studies on which the present study is based | 21-22 | The present research used publicly available summary data, where no extra ethical approval is required. This work was supported by Samarbeidsorganet Helse Midt-Norge and the Norwegian University of Science and Technology, NTNU. The funders had no role in study design, data collection, data analysis, data interpretation, writing of the report, or in the decision to submit the article for publication. |
| 19 | Data and data sharing | Provide the data used to perform all analyses or report where and how the data can be accessed and reference these sources in the article. Provide the statistical code needed to reproduce the results in the article, or report whether the code is publicly accessible and if so, where |  | Exposure: Additional File 2 Additional text, Table 2 and Additional File 2 Table S7 for genetic instruments used as exposure.  Table 2 and Additional File 3 Table S5 (meta-analyses results) for genetic instruments used as outcome. |
| 20 | Conflicts of Interest | All authors should declare all potential conflicts of interest | 21 | The authors declare that they have no competing interests. |

This checklist is copyrighted by the Equator Network under the Creative Commons Attribution 3.0 Unported (CC BY 3.0) license.
