# Supplementary material for: Circulating levels of micronutrients and risk of infections: a Mendelian randomization study
Source: BMC Med. 2023 Mar 8;21:84. doi: 10.1186/s12916-023-02780-3 (PMC9993583; doi:10.1186/s12916-023-02780-3)
Supplement: Supplementary file 2 — Additional file 2: Additional Text – Exposure GWAS cohorts. Fig. S1. Scatter plot of secondary MR analysis of copper as risk factors on the risk of gastrointestinal infections. Table S2. ICD-10 codes for gastrointestinal infections in UK Biobank and FinnGen R6. Table S3. ICD-10 codes for pneumonia in UK Biobank, and FinnGen. Table S4. ICD-10 codes for urinary tract infection UK Biobank, and FinnGen. Table S6. Power calculations. Table S7. Genetic variants used as exposure for Mendelian randomization analyses. Table S8. Main mendelian randomization analyses of micronutrients as risk factors on the risk of gastrointestinal infections. Table S9. Main mendelian randomization analyses of micronutrients as risk factors on the risk of pneumonia. Table S10. Main mendelian randomization analyses of micronutrients as risk factors on the risk of urinary tract infections. Table S11. Secondary mendelian randomization analyses of micronutrients as risk factors on the risk of gastrointestinal infections, pneumonia and urinary tract infections suggestive-significant genetic instruments. Table S12. Secondary mendelian randomization analyses of copper as risk factors on the risk of gastrointestinal infections, where rs12582659 was removed. Table S21. IVW MR regression results for the leave one SNP out analysis in the Mendelian randomization analyses of micronutrients. [file 12916_2023_2780_MOESM2_ESM.zip › HeleneMarieFlatby_Additional_file_2_BMED-D-22-02781R1R2.docx]

**Additional file 2**

**Circulating levels of micronutrients and risk of infections: A Mendelian randomization study**

Helene M Flatby, MSc^1,2*^ (helene.flatby@ntnu.no) http://orcid.org/0000-0002-5700-020X

Anuradha Ravi, PhD^1,2^ (anuradha.ravi@ntnu.no) https://orcid.org/0000-0001-5668-0090

Jan K Damås, MD PhD^1,3,4^ (jan.k.damas@ntnu.no) http://orcid.org/0000-0003-4268-671X

Erik Solligård, MD PhD^1,2^ (erik.solligard@ntnu.no) http://orcid.org/0000-0001-6173-3580

Tormod Rogne, MD PhD^5,1^ (tormod.rogne@yale.edu) http://orcid.org/0000-0002-9581-7384

* Corresponding author

1. Gemini Center for Sepsis Research, Department of Circulation and Medical Imaging, NTNU, Norwegian University of Science and Technology, Trondheim, Norway
2. Clinic of Anaesthesia and Intensive Care, St. Olavs Hospital, Trondheim University Hospital, Trondheim, Norway
3. Centre of Molecular Inflammation Research, Department of Clinical and Molecular Medicine, NTNU, Norwegian University of Science and Technology, Trondheim, Norway
4. Department of Infectious Diseases, St. Olavs Hospital, Trondheim University Hospital, Trondheim, Norway
5. Department of Chronic Disease Epidemiology and Center for Perinatal, Pediatric and Environmental Epidemiology, Yale School of Public Health, New Haven, CT, USA

**Corresponding author:**

Helene Marie Flatby

Department of Circulation and Medical Imaging, NTNU

Prinsesse Kristinas gate 3, Akutten og Hjerte-lunge-senteret, 3. etg

Trondheim 7491, Norway

Email: helene.flatby@ntnu.no

Phone: +47 472 65 442

ORCiD: <https://orcid.org/0000-0002-5700-020X>

**Short title:** Micronutrients and risk of infections

**Keywords:** Micronutrients; Mendelian randomization; Gastrointestinal infections; Infections; Copper

**Additional Text: Exposure GWAS cohorts**

**Calcium genetic instruments**

The genetic instruments used for calcium were extracted from a meta-analysis conducted using 39,400 individuals from 17 population-based cohorts in the discovery stage and 21,679 individuals in the replication stage, totaling approximately 61,079 individuals. The extracted SNP was from either the discovery (n = 39,400) cohort or the meta-analysis. The mean concentration of serum calcium level was 9.47 mg/dL (SD 0.52). The covariates included in the GWAS were age, sex, and study-specific covariates, if needed, such as principal components and study center.

**Copper genetic instruments**

The genetic instruments used for copper were extracted from a GWAS conducted using 2,603 twins and families from the Queensland Institute of Medical Research (QIMR). The mean concentration of erythrocyte Cu was 43.7 nmol/g (SD 12.15). Estimates for genetic associations with log-transformed standardized residuals of copper were adjusted for sex and age via an additive model accounting for within-family relatedness, where the effect was measured in SD. We conducted secondary analyses (using *r^2^* < 0.01 within 10,000 kb windows and *P* ≤ 5E-06), where we used the summary statistics from Evans et al. in addition to results from a meta-analysis conducted by Jäger et al. with 6,937 individuals. The meta-analysis by Jäger et al. was performed using a weighted z-score approach, meaning that beta estimates for the associations with copper and the effect sizes from the MR analysis don't have interpretable units since the studies only share z-scores. However, the results are valuable in effect direction. Because the reported effects were expressed in Z-score unit per effect allele, we converted the Z-scores to beta and standard error using the same formula as Jäger et al. : beta=z-score/sqrt(N) × 1/ sqrt (EAF(1-EAF)) and SE=beta/z-score, where EAF is the allele frequency of the effect allele, N is the sample size of the meta-analysis and z-score the corresponding (and provided) z-score.

**Iron genetic instruments**

The genetic instruments used for iron were extracted from a meta-analysis using results from 129,305 individuals from Iceland (deCODE genetics) and 45,438 from the UK (INTERVAL study), totaling 174,743 individuals. The mean concentration of serum iron was 15.88 µmol/L (SD 6.84). The meta-analysis was performed using fixed-effect inverse variance weighted method. The GWAS conducted using deCODE was adjusted for sex, age, and county of birth, while the GWAS conducted using the INTERVAL study was adjusted for sex, age, and principal components.

**Magnesium genetic instruments**

The genetic instruments used for magnesium were extracted from a meta-analysis using approximately 15,366 individuals from the Cohorts for Heart and Aging Research in Genomic Epidemiology (CHARGE) consortium, which consisted of 8,122 individuals from the Atherosclerosis Risk in Communities (ARIC) Study, 2,866 individuals from the Framingham Heart Study (FHS) and 4,378 individuals from the Rotterdam Study (RS). The mean concentration of serum magnesium was 0.84 mmol/L (SD 0.09). The individual GWASs were adjusted for age, sex, and study center (if applicable), and meta-analyses were performed using inverse-variance weighted fixed-effects models.

**Selenium genetic instruments**

The genetic instruments used for selenium were extracted from a meta-analysis using 2,603 individuals from QIMR study and 2,874 from The Avon Longitudinal Study of Parents and Children (ALSPAC) cohort, totaling 5,477. Estimates for genetic associations with log-transformed standardized residuals of selenium were adjusted for sex and age via an additive model accounting for within-family relatedness, where the effect was measured in SD. For SNPs with *P* ≤ 5E-06 used in the secondary analyses, we only had access to results from individual cohorts QIMR and ALSPAC.

**Zinc genetic instruments**

The genetic instruments used for zinc were extracted from a GWAS conducted using 2,603 individuals from the QIMR cohort. Estimates for genetic associations with log-transformed standardized residuals of zinc were adjusted for sex and age via an additive model accounting for within-family relatedness, where the effect was measured in SD.

**Beta-carotene genetic instruments**

The genetic instruments used for beta-carotene were extracted from a meta-analysis conducted using 1,190 individuals from the Invecchiare in Chianti (InCHIANTI) study, 576 individuals from Women's Health and Aging Study (WHAS), and 2,126 individuals from the α-Tocopherol, β-Carotene Cancer Prevention (ATBC) Study, totaling approximately 3,892 individuals. The mean concentration serum/plasma level of beta-carotene was 0.41 μmol/L (SD 0.27). The meta-analysis was performed across different studies by weighting each with an inverse variance method. The individual GWASs were adjusted for age and sex. In the harmonizing process of the two-sample MR analyses for beta-carotene, we identified that the genetic variants were ambiguous, and we could, therefore, not perform power calculation analysis using P ≤ 5E-06 for beta-carotene.

**Folate genetic instruments**

The genetic instruments used for folate were extracted from GWAS conducted using 28,913 Icelanders. The mean concentration of serum folate was 22.6 pmol/l (SD 11.33) for Icelanders. The GWAS was adjusted for sex, year of birth, and age at measurement.

**Vitamin B6 genetic instruments**

The genetic instruments used for vitamin B6 were extracted from a meta-analysis using 1,658 individuals from the Nurses' Health Study (NHS) Cancer Genetic Markers of Susceptibility (CGEMS), 1,647 from Framingham-SNP-Health Association Resource (SHARe) Women, and 1,458 from SHARe men, totaling approximately 4,763 individuals. The mean concentration of plasma vitamin B6 was 81.94 pmol/ml (SD 86.03). A fixed-effect model was used to conduct the meta-analysis. The CGEMS GWAS was adjusted for age and principal components, while the SHARe data was adjusted for age.

**Vitamin B12 genetic instruments**

The genetic instruments used for vitamin B12 were extracted from a meta-analysis using approximately 25,960 individuals from Icelandic, 5,481 individuals from Danish – inter99, and 2,812 individuals from Danish -Health 2006 study, totaling 45,575. The mean concentration of serum vitamin B12 was 385.07 pmol/l (SD 221.09). The data were combined using fixed-effect meta-analyses based on *P* values and direction of effect adjusted for the number of individuals in each sample. We only had access to *P* ≤ 5E-06 SNPs from the Icelandic data.

**Vitamin C genetic instruments**

The genetic instruments used for vitamin C were extracted from a meta-analysis of 52,018 individuals using four cohort studies, **Fenland, European Prospective Investigation into Cancer (EPIC) - Norfolk, EPIC-InterAct, and EPIC-** **Cardiovascular disease (CVD).** The mean concentration of plasma vitamin C was 50.1 umol/l (SD 23.1). The meta-analysis of GWAS results was conducted by combining beta coefficients and SE using inverse variance-weighted fixed-effect meta-analysis across the participating studies. The individual GWASs were adjusted for age, sex, study center (where appropriate), and the first ten principal components.

**Vitamin D genetic instruments**

The genetic instruments used for vitamin D were extracted from GWAS conducted using 401,460 individuals from UK Biobank. The mean concentration of serum vitamin D was 70.0 nmol/L (SD 34.7). The GWAS was performed on standardized log-transformed vitamin D expressed in nmol/L, adjusted for age, sex, season of measurement, and vitamin D supplementation. For the secondary analysis, we only had access to *P* ≤ 5E-06 SNPs from a meta-analysis, where one of the included GWAS was adjusted for BMI, which might introduce collider bias.

**Fig. S1.** Scatter plot of secondary MR analysis of copper as risk factors on the risk of gastrointestinal infections

*Legend:* Scatter plot of the secondary MR-analysis (*r^2^* < 0.01 within 10,000 kb windows and *P* ≤ 5E-06) using Cu from Evans et al. and meta-analysis summary-statistics for gastrointestinal infection.

| **Table S2.** ICD-10 codes for gastrointestinal infections in UK Biobank and FinnGen R6. | | |
| --- | --- | --- |
| **Code** | **Description** | **Cohort^a^** |
| A00 | Cholera | UKB, FinnGen R6 |
| A00.0 | Cholera due to Vibrio cholerae 01, biovar cholerae | UKB |
| A00.1 | Cholera due to Vibrio cholerae 01, biovar eltor | UKB |
| A00.9 | Cholera, unspecified | UKB |
| A01 | Typhoid and paratyphoid fevers | UKB, FinnGen R6 |
| A01.1 | Paratyphoid fever A | UKB |
| A01.2 | Paratyphoid fever B | UKB |
| A01.3 | Paratyphoid fever C | UKB |
| A01.4 | Paratyphoid fever, unspecified | UKB |
| A02 | Other salmonella infections | FinnGen R6 |
| A03 | Shigellosis | FinnGen R6 |
| A04 | Other bacterial intestinal infections | FinnGen R6 |
| A05 | Other bacterial foodborn intoxications, not elsewhere classified | FinnGen R6 |
| A05.9 | Bacterial foodborne intoxication, unspecified | UKB |
| A06 | Amoebiasis | UKB, FinnGen R6 |
| A06.2 | Amoebic nondysenteric colitis | UKB |
| *Continued on next page* | | |
| **Table S2. *Continued*** | | |
| A06.3 | Amoeboma of intestine | UKB |
| A06.4 | Amoebic liver abscess | UKB |
| A06.5 | Amoebic lung abscess | UKB |
| A06.6 | Amoebic brain abscess | UKB |
| A06.7 | Cutaneous amoebiasis | UKB |
| A06.8 | Amoebic infection of other sites | UKB |
| A06.9 | Amoebiasis, unspecified | UKB |
| A07 | Other protozoal intestinal diseases | UKB, FinnGen R6 |
| A07.3 | Isosporiasis | UKB |
| A07.9 | Protozoal intestinal disease, unspecified | UKB |
| A08 | Viral and other specified intestinal infections | FinnGen R6 |
| A09 | Other gastroenteritis and colitis of infectious and unspecified origin | UKB, FinnGen R6 |
| A21.3 | Gastrointestinal tularaemia | UKB |
| A22.2 | Gastrointestinal anthrax | UKB |
| ^a^UK Biobank denoted UKB | | |

| **Table S3.** ICD-10 codes for pneumonia in UK Biobank, and FinnGen. | | |
| --- | --- | --- |
| **Code** | **Description** | **Cohort^a^** |
| A20.2 | Pneumonic plague | UKB |
| A21.2 | Pulmonary tularaemia | UKB |
| A22.1 | Pulmonary anthrax | UKB |
| A31.0 | Pulmonary mycobacterial infection | UKB |
| A42.0 | Pulmonary actinomycosis | UKB |
| A43.0 | Pulmonary nocardiosis | UKB |
| A48.1 | Legionnaires disease | UKB |
| A78 | Q fever | UKB |
| B96.0 | Mycoplasma pneumoniae [M. pneumoniae] as the cause of diseases classified to other chapters | UKB |
| B96.1 | Klebsiella pneumoniae [K. pneumoniae] as the cause of diseases classified to other chapters | UKB |
| J13 | Pneumonia due to streptococcus pneumonia | FinnGen R6 |
| J14 | Pneumonia due to Hemophilus influenzae | UKB, FinnGen R6 |
| J15 | Bacterial pneumonia, not elsewhere classified | FinnGen R6 |
| J15.0 | Pneumonia due to Klebsiella pneumoniae | UKB |
| *Continued on next page* | | |
| **Table S3. *Continued*** | | |
| J15.2 | Pneumonia due to staphylococcus | UKB |
| J15.3 | Pneumonia due to streptococcus, group B | UKB |
| J15.4 | Pneumonia due to other streptococci | UKB |
| J15.5 | Pneumonia due to Escherichia coli | UKB |
| J15.6 | Pneumonia due to other aerobic Gram-negative bacteria | UKB |
| J15.7 | Pneumonia due to Mycoplasma pneumoniae | UKB |
| J15.8 | Other bacterial pneumonia | UKB |
| J15.9 | Bacterial pneumonia, unspecified | UKB |
| J16.0 | Chlamydial pneumonia | UKB |
| J17.0 | Pneumonia in bacterial diseases classified elsewhere | FinnGen R6 |
| ^a^UK Biobank denoted UKB | | |

| **Table S4.** ICD-10 codes for urinary tract infection UK Biobank, and FinnGen. | | |
| --- | --- | --- |
| **Code** | **Description** | **Cohort^a^** |
| N36 | Other disorders of urethra | FinnGen R6 |
| N39 | Other disorders of urinary system | FinnGen R6 |
| N39.0 | Urinary tract infection, site not specified | UKB |
| ^a^UK Biobank denoted UKB | | |

| **Table S6.** Power calculations. | | | | | | | | | | | | |
| --- | --- | --- | --- | --- | --- | --- | --- | --- | --- | --- | --- | --- |
|  |  | **Gastrointestinal infections** | | |  | **Pneumonia** | | |  | **Urinary tract infections** | | |
| **Exposure** |  | Main analysis^a^ | Secondary analysis^b^ | |  | Main analysis^a^ | Secondary analysis^b^ | |  | Main analysis^a^ | Secondary analysis^b^ | |
| Ca |  | 11 | 12 | |  | 8 | 8 | |  | 10 | 12 | |
| Cu |  | 97 | QIMR | 100 |  | 76 | QIMR | 100 |  | 96 | QIMR | 100 |
|  |  | 100 | Jäger et al. | 100 |  | 100 | Jäger et al. | 90 |  | 100 | Jäger et al. | 99 |
| Fe |  | 89 | 99 | |  | 60 | 84 | |  | 86 | 98 | |
| Mg |  | 5 | 6 | |  | 5 | 5 | |  | 5 | 5 | |
| Se |  | 81 | QIMR | 100 |  | 51 | QIMR | 98 |  | 77 | QIMR | 100 |
|  |  |  | ALSPA | 100 |  | 98 | ALSPA | 98 |  | 100 | ALSPA | 100 |
| Zn |  | 96 | 100 | |  | 75 | 96 | |  | 95 | 100 | |
| Beta carotene |  | 49 | - | |  | 78 | - | |  | 96 | - | |
| Folate |  | 29 | 32 | |  | 16 | 32 | |  | 27 | 32 | |
| Vitamin B6 |  | 31 | - | |  | 18 | - | |  | 30 | - | |
| Vitamin B12 |  | 97 | 97 | |  | 76 | 97 | |  | 96 | 97 | |
| Vitamin C |  | 71 | 79 | |  | 42 | 79 | |  | 68 | 79 | |
| Vitamin D |  | 73 | 81 | |  | 43 | 50 | |  | 69 | 77 | |
| Missing data denoted -. Results are presented in %.  ^a^ SNPs with *r^2^* < 0.001 within 10,000 kb windows and *P* ≤ 5E-08 were used for the main analyses  ^b^ SNPs with *r^2^* < 0.01 within 10,000 kb and *P* ≤ 5E-06 were used for the secondary analyses.  Abbreviations: ALSPA, Avon Longitudinal Study of Parents and Children; Ca, Calcium; Cu, Copper; Fe, Iron; Mg, Magnesium;  QIMR, Queensland Institute of Medical Research; Selenium; Zn, Zinc. | | | | | | | | | | | | |

| **Table S7.** Genetic variants used as exposure for Mendelian randomization analyses. | | | | | | | | | | | | | | | |
| --- | --- | --- | --- | --- | --- | --- | --- | --- | --- | --- | --- | --- | --- | --- | --- |
| **Exposure** | **Analysis^a^** | **rsID** | **Gene^b^** | **CHR** | **BP^c^** | **EAF** | **EA** | **OA** | **OR^d^** | **Beta** | **StdErr** | ***P* value** | **R^2^** | **F statistics** | **N** |
| Cu | Main | rs1175550 | *SMIM1* | 1 | 3774964 | 0.74 | A | G | 0.82 | -0.20 | 0.032 | 5.03E-10 | 0.0152 | 40 | 2603 |
|  |  | rs2769264 | *SELENBP1* | 1 | 151372265 | 0.18 | G | T | 1.37 | 0.31 | 0.034 | 2.63E-20 | 0.0284 | 76 | 2603 |
|  | Secondary  Evans et al. | rs10014072 | *ANK2* | 4 | 113027634 | 0.33 | A | G | 1.18 | 0.16 | 0.034 | 1.13E-06 | 0.0119 | 31 | 2603 |
|  |  | rs12153606 | *CTD-2232E5.2* | 5 | 85287951 | 0.83 | G | T | 1.17 | 0.16 | 0.034 | 2.50E-06 | 0.0072 | 19 | 2603 |
|  |  | rs3857536 | *RNU7-66P* | 6 | 66219155 | 0.48 | C | T | 1.14 | 0.13 | 0.028 | 4.08E-06 | 0.0083 | 22 | 2603 |
|  |  | rs12582659 | *RP11-114H23.1* | 12 | 75670748 | 0.06 | C | T | 3.53 | 1.26 | 0.270 | 2.86E-06 | 0.1780 | 563 | 2603 |
|  | Secondary  Jäger et al.^e^ | rs114415211 | *POGZ* | 1 | 151466582 | 0.02 | A | G | 1.60 | 0.47 | 0.094 | 5.09E-07 | 0.0073 | 51 | 6937 |
|  |  | rs116071814 | *LOXL2* | 8 | 23352269 | 0.06 | T | C | 1.27 | 0.24 | 0.051 | 4.60E-06 | 0.0061 | 42 | 6937 |
|  |  | rs2050913 | *FAF2P1* | 10 | 91773911 | 0.34 | T | C | 1.13 | 0.12 | 0.025 | 2.84E-06 | 0.0063 | 44 | 6937 |
|  |  | rs1175552 | *CCDC27* | 1 | 3771175 | 0.24 | A | C | 1.16 | 0.15 | 0.028 | 2.14E-07 | 0.0078 | 54 | 6937 |
|  |  | rs6467617 | *AC015987.1* | 7 | 135906335 | 0.07 | T | C | 1.26 | 0.23 | 0.049 | 2.25E-06 | 0.0065 | 45 | 6937 |
|  |  | rs17564336 | *SELENBP1* | 1 | 151376698 | 0.30 | T | G | 0.80 | -0.22 | 0.026 | 1.79E-17 | 0.0209 | 148 | 6937 |
|  |  | rs34951015 | *CP* | 3 | 149208528 | 0.10 | A | G | 1.28 | 0.25 | 0.040 | 9.57E-10 | 0.0108 | 76 | 6937 |
| Fe | Main | rs1800562 | *HFE* | 6 | 26092913 | 0.07 | A | G | 1.31 | 0.27 | 0.008 | 1.00E-200 | 0.0092 | 1623 | 174743 |
|  |  | rs855791 | *TMPRSS6* | 22 | 37066896 | 0.43 | A | G | 0.84 | -0.17 | 0.005 | 1.00E-200 | 0.0142 | 2513 | 174743 |
|  |  | rs7385804 | *TFR2* | 7 | 100638347 | 0.37 | C | A | 0.94 | -0.06 | 0.004 | 9.42E-43 | 0.0015 | 265 | 174743 |
|  |  | rs9399136 | *HBS1L* | 6 | 135081201 | 0.26 | C | T | 1.06 | 0.06 | 0.005 | 1.08E-36 | 0.0012 | 218 | 174743 |
|  |  | rs4854760 | *SRPRB* | 3 | 133779897 | 0.31 | G | A | 1.05 | 0.05 | 0.004 | 5.67E-33 | 0.0012 | 211 | 174743 |
|  |  | rs35945185 | *LEPR* | 1 | 65671556 | 0.37 | A | G | 1.03 | 0.03 | 0.004 | 1.54E-13 | 0.0004 | 78 | 174743 |
|  |  | rs13007705 | *ERFE* | 2 | 238160555 | 0.43 | T | C | 1.03 | 0.03 | 0.004 | 2.01E-12 | 0.0004 | 72 | 174743 |
|  |  | rs2005682 | *FFAR2* | 19 | 35456759 | 0.31 | T | A | 0.97 | -0.03 | 0.004 | 2.37E-11 | 0.0004 | 62 | 174743 |
|  |  | rs12718598 | *IKZF1* | 7 | 50360747 | 0.46 | C | T | 1.03 | 0.03 | 0.004 | 3.69E-11 | 0.0004 | 63 | 174743 |
| *Continued on next page* | | | | | | | | | | | | | | | |
|  |  |  |  |  |  |  |  |  |  |  |  |  |  |  |  |
| **Table S7. *Continued*** | | | | | | | | | | | | | | | |
| Fe | Main | rs2228145 | *IL6R* | 1 | 154454494 | 0.41 | C | A | 1.03 | 0.03 | 0.004 | 8.42E-11 | 0.0003 | 57 | 174743 |
|  |  | rs77262773 | *ABCA5* | 17 | 69253570 | 0.03 | T | C | 1.08 | 0.08 | 0.013 | 9.54E-10 | 0.0003 | 58 | 174743 |
|  |  | rs7630745 | *SLC25A26* | 3 | 66376605 | 0.36 | C | T | 1.03 | 0.03 | 0.004 | 2.09E-09 | 0.0003 | 50 | 174743 |
|  |  | rs57659670 | *DUOX2* | 15 | 45106240 | 0.08 | C | T | 0.96 | -0.04 | 0.007 | 1.08E-08 | 0.0002 | 43 | 174743 |
|  | Secondary | rs1799945 | *HFE* | 6 | 26090951 | 0.14 | G | C | 1.19 | 0.17 | 0.006 | 1.26E-187 | 0.0068 | 1202 | 174743 |
|  |  | rs2844822 | *-* | 6 | 29868870 | 0.12 | C | G | 0.97 | -0.03 | 0.006 | 5.02E-08 | 0.0002 | 36 | 174743 |
|  |  | rs116019440 | *HIST1H1C* | 6 | 26061092 | 0.04 | A | T | 0.94 | -0.06 | 0.011 | 5.04E-08 | 0.0003 | 47 | 174743 |
|  |  | rs113130862 | *ZBED9* | 6 | 28617865 | 0.02 | G | A | 0.93 | -0.07 | 0.014 | 5.04E-08 | 0.0003 | 46 | 174743 |
|  |  | rs7251303 | *FAM187B* | 19 | 35231841 | 0.38 | A | C | 1.02 | 0.02 | 0.004 | 5.14E-08 | 0.0002 | 41 | 174743 |
|  |  | rs2237227 | *HIST1H1T* | 6 | 26104667 | 0.05 | A | C | 0.95 | -0.05 | 0.009 | 5.20E-08 | 0.0002 | 42 | 174743 |
|  |  | rs550629667 | *ZNF311* | 6 | 28994615 | 0.02 | T | C | 1.08 | 0.08 | 0.015 | 5.22E-08 | 0.0003 | 46 | 174743 |
|  |  | rs9274712 | *HLA-DQB1* | 6 | 32669521 | 0.27 | T | C | 1.02 | 0.02 | 0.004 | 5.31E-08 | 0.0002 | 41 | 174743 |
|  |  | rs140850749 | *GUSBP2* | 6 | 26891662 | 0.04 | G | C | 0.94 | -0.06 | 0.011 | 5.39E-08 | 0.0003 | 51 | 174743 |
|  |  | rs1265184 | *POU5F1* | 6 | 31183985 | 0.02 | A | G | 1.09 | 0.08 | 0.015 | 5.50E-08 | 0.0002 | 41 | 174743 |
|  |  | rs10740134 | *REEP3* | 10 | 63555673 | 0.48 | T | C | 0.98 | -0.02 | 0.004 | 5.82E-08 | 0.0002 | 41 | 174743 |
|  |  | rs80067878 | *-* | 6 | 25574997 | 0.01 | T | C | 1.11 | 0.10 | 0.019 | 5.99E-08 | 0.0002 | 42 | 174743 |
|  |  | rs760520 | *NCF4-AS1* | 22 | 36851236 | 0.38 | G | C | 0.98 | -0.02 | 0.004 | 6.72E-08 | 0.0003 | 48 | 174743 |
|  |  | rs146700177 | *RBFOX2* | 22 | 35826309 | 0.10 | C | T | 0.96 | -0.04 | 0.007 | 6.93E-08 | 0.0002 | 42 | 174743 |
|  |  | rs72836134 | *CASC15* | 6 | 22218001 | 0.02 | G | A | 1.08 | 0.08 | 0.015 | 8.09E-08 | 0.0002 | 43 | 174743 |
|  |  | rs73163080 | *ENTHD1* | 22 | 39891930 | 0.05 | A | T | 0.95 | -0.05 | 0.010 | 8.57E-08 | 0.0003 | 44 | 174743 |
|  |  | rs187570181 | *NOTCH4* | 6 | 32196810 | 0.06 | T | C | 0.95 | -0.05 | 0.009 | 9.17E-08 | 0.0003 | 49 | 174743 |
|  |  | rs2284009 | *CACNG2* | 22 | 36682504 | 0.11 | T | C | 0.97 | -0.03 | 0.006 | 1.02E-07 | 0.0002 | 41 | 174743 |
|  |  | rs149906368 | *HIST1H3E* | 6 | 26229080 | 0.02 | T | C | 0.93 | -0.08 | 0.015 | 1.07E-07 | 0.0003 | 48 | 174743 |
|  |  | rs2294915 | *PNPLA3* | 22 | 43945024 | 0.24 | T | C | 1.03 | 0.02 | 0.005 | 1.08E-07 | 0.0002 | 40 | 174743 |
| *Continued on next page* | | | | | | | | | | | | | | | |
|  |  |  |  |  |  |  |  |  |  |  |  |  |  |  |  |
| **Table S7. *Continued*** | | | | | | | | | | | | | | | |
| Fe | Secondary | rs5756391 | *SCUBE1* | 22 | 36902302 | 0.41 | A | G | 1.02 | 0.02 | 0.004 | 1.16E-07 | 0.0002 | 40 | 174743 |
|  |  | rs73161899 | *SCUBE1* | 22 | 37264661 | 0.03 | A | C | 0.94 | -0.06 | 0.011 | 1.24E-07 | 0.0002 | 36 | 174743 |
|  |  | rs739936 | *PLEKHA7* | 11 | 16783679 | 0.21 | T | C | 0.97 | -0.03 | 0.005 | 1.34E-07 | 0.0002 | 38 | 174743 |
|  |  | rs77811187 | *CSF2RB* | 22 | 36933594 | 0.02 | A | G | 0.93 | -0.07 | 0.013 | 1.53E-07 | 0.0002 | 39 | 174743 |
|  |  | rs61804209 | *Y_RNA* | 1 | 161696843 | 0.15 | G | A | 1.03 | 0.03 | 0.006 | 1.67E-07 | 0.0002 | 42 | 174743 |
|  |  | rs12190171 | *HIST1H2BA* | 6 | 25722740 | 0.03 | C | T | 0.95 | -0.06 | 0.011 | 1.75E-07 | 0.0002 | 35 | 174743 |
|  |  | rs483792 | *ZFP57* | 6 | 23964253 | 0.27 | A | C | 1.02 | 0.02 | 0.005 | 1.80E-07 | 0.0002 | 38 | 174743 |
|  |  | rs387628 | *FAM65B* | 6 | 24951581 | 0.37 | A | G | 0.98 | -0.02 | 0.004 | 2.04E-07 | 0.0002 | 39 | 174743 |
|  |  | rs116153196 | *ZFP57* | 6 | 28001658 | 0.02 | A | G | 1.08 | 0.08 | 0.015 | 2.05E-07 | 0.0002 | 36 | 174743 |
|  |  | rs2158799 | *JAZF1-AS1* | 7 | 28237488 | 0.40 | C | G | 0.98 | -0.02 | 0.004 | 2.73E-07 | 0.0002 | 37 | 174743 |
|  |  | rs9260480 | *HLA-W* | 6 | 29952386 | 0.08 | C | T | 1.04 | 0.04 | 0.008 | 2.78E-07 | 0.0003 | 45 | 174743 |
|  |  | rs550852967 | *SCUBE1* | 22 | 36415265 | 0.01 | A | G | 0.92 | -0.09 | 0.017 | 2.89E-07 | 0.0002 | 35 | 174743 |
|  |  | rs28929474 | *SERPINA1* | 14 | 94378610 | 0.01 | T | C | 1.10 | 0.09 | 0.018 | 3.66E-07 | 0.0001 | 24 | 174743 |
|  |  | rs12110459 | *C6orf222* | 6 | 36319912 | 0.08 | A | T | 1.04 | 0.04 | 0.008 | 3.99E-07 | 0.0002 | 36 | 174743 |
|  |  | rs55709272 | *IL1RN* | 2 | 113109711 | 0.49 | C | T | 0.98 | -0.02 | 0.004 | 4.16E-07 | 0.0002 | 36 | 174743 |
|  |  | rs111265204 | *ZNF322* | 6 | 26638189 | 0.01 | A | G | 0.90 | -0.11 | 0.022 | 4.49E-07 | 0.0002 | 35 | 174743 |
|  |  | rs59755875 | *SSTR3* | 22 | 37215644 | 0.15 | G | T | 0.97 | -0.03 | 0.006 | 4.86E-07 | 0.0002 | 38 | 174743 |
|  |  | rs146139049 | *snoU13* | 22 | 37788351 | 0.03 | A | G | 1.06 | 0.06 | 0.012 | 5.64E-07 | 0.0002 | 38 | 174743 |
|  |  | rs12143966 | *NLRP3* | 1 | 247438055 | 0.38 | A | G | 0.98 | -0.02 | 0.004 | 6.35E-07 | 0.0002 | 35 | 174743 |
|  |  | rs41271815 | *CARMIL1* | 6 | 25472509 | 0.06 | T | C | 0.96 | -0.04 | 0.008 | 6.92E-07 | 0.0002 | 33 | 174743 |
|  |  | rs116116350 | *KRT18P35* | 3 | 133919454 | 0.01 | C | G | 0.89 | -0.11 | 0.023 | 7.19E-07 | 0.0002 | 28 | 174743 |
|  |  | rs213206 | *RPS18* | 6 | 33273366 | 0.30 | G | C | 1.02 | 0.02 | 0.004 | 8.02E-07 | 0.0002 | 33 | 174743 |
|  |  | rs73181000 | *IGF1* | 12 | 102467405 | 0.05 | A | G | 1.05 | 0.05 | 0.010 | 8.35E-07 | 0.0002 | 40 | 174743 |
|  |  | rs12407066 | *LINC01344* | 1 | 182210827 | 0.26 | A | C | 0.98 | -0.02 | 0.005 | 8.95E-07 | 0.0002 | 34 | 174743 |
| *Continued on next page* | | | | | | | | | | | | | | | |
|  |  |  |  |  |  |  |  |  |  |  |  |  |  |  |  |
| **Table S7. *Continued*** | | | | | | | | | | | | | | | |
| Fe | Secondary | rs114257178 | *LL22NC01-81G9.3* | 22 | 36965961 | 0.10 | T | A | 0.97 | -0.04 | 0.007 | 8.98E-07 | 0.0002 | 40 | 174743 |
|  |  | rs9607414 | *IL2RB* | 22 | 37121657 | 0.30 | T | C | 0.98 | -0.02 | 0.004 | 9.36E-07 | 0.0002 | 34 | 174743 |
|  |  | rs1233397 | *GABBR1* | 6 | 29577938 | 0.24 | G | A | 0.98 | -0.02 | 0.005 | 9.51E-07 | 0.0002 | 34 | 174743 |
|  |  | rs983828 | *PRRT4* | 7 | 128366341 | 0.26 | G | A | 0.98 | -0.02 | 0.005 | 9.56E-07 | 0.0002 | 34 | 174743 |
|  |  | rs140247848 | *TMEM184B* | 22 | 38273009 | 0.05 | A | G | 1.05 | 0.05 | 0.010 | 9.84E-07 | 0.0002 | 40 | 174743 |
|  |  | rs2042919 | *-* | 19 | 7899064 | 0.25 | G | A | 0.98 | -0.02 | 0.005 | 1.01E-06 | 0.0002 | 33 | 174743 |
|  |  | rs12705056 | *MYH16* | 7 | 99233733 | 0.13 | T | C | 1.03 | 0.03 | 0.006 | 1.11E-06 | 0.0002 | 31 | 174743 |
|  |  | rs4817984 | *-* | 21 | 39093140 | 0.31 | A | C | 1.02 | 0.02 | 0.004 | 1.19E-06 | 0.0002 | 34 | 174743 |
|  |  | rs1573673 | *IL2RB* | 22 | 37172630 | 0.09 | T | C | 1.03 | 0.03 | 0.007 | 1.23E-06 | 0.0002 | 31 | 174743 |
|  |  | rs1223763 | *RP11-53A1.3* | 1 | 214160903 | 0.23 | T | G | 1.02 | 0.02 | 0.005 | 1.74E-06 | 0.0002 | 34 | 174743 |
|  |  | rs62230766 | *TEX33* | 22 | 37009630 | 0.18 | T | C | 0.97 | -0.03 | 0.005 | 1.76E-06 | 0.0002 | 34 | 174743 |
|  |  | rs9607411 | *TMPRSS6* | 22 | 37083824 | 0.02 | C | T | 1.08 | 0.07 | 0.015 | 1.76E-06 | 0.0002 | 30 | 174743 |
|  |  | rs7216369 | *ARHGAP44* | 17 | 12956998 | 0.09 | T | C | 0.97 | -0.03 | 0.007 | 1.88E-06 | 0.0002 | 33 | 174743 |
|  |  | rs4729627 | *MUC3A* | 7 | 100952788 | 0.25 | A | C | 0.98 | -0.02 | 0.005 | 2.08E-06 | 0.0002 | 31 | 174743 |
|  |  | rs2613522 | *TMC6* | 17 | 78123984 | 0.28 | G | A | 0.98 | -0.02 | 0.004 | 2.09E-06 | 0.0002 | 32 | 174743 |
|  |  | rs74338506 | *FYN* | 6 | 111733099 | 0.02 | A | G | 0.94 | -0.06 | 0.014 | 2.14E-06 | 0.0002 | 29 | 174743 |
|  |  | rs141899063 | *TBC1D22B* | 6 | 31234077 | 0.01 | C | T | 1.10 | 0.09 | 0.019 | 2.40E-06 | 0.0002 | 31 | 174743 |
|  |  | rs2043192 | *STK25* | 2 | 235297146 | 0.44 | G | A | 0.98 | -0.02 | 0.004 | 2.58E-06 | 0.0002 | 32 | 174743 |
|  |  | rs4064993 | *SLC25A37* | 8 | 23523997 | 0.44 | T | A | 1.02 | 0.02 | 0.004 | 2.75E-06 | 0.0002 | 31 | 174743 |
|  |  | rs74691901 | *ZFP57* | 6 | 25900665 | 0.01 | G | A | 0.91 | -0.10 | 0.021 | 2.77E-06 | 0.0002 | 39 | 174743 |
|  |  | rs116422639 | *PRL* | 6 | 22301367 | 0.02 | T | C | 1.06 | 0.06 | 0.013 | 2.82E-06 | 0.0001 | 25 | 174743 |
|  |  | rs10261327 | *SP4* | 7 | 21423210 | 0.44 | T | C | 1.02 | 0.02 | 0.004 | 2.92E-06 | 0.0002 | 31 | 174743 |
|  |  | rs77943558 | *TTLL5* | 14 | 75772296 | 0.02 | T | C | 1.08 | 0.08 | 0.017 | 2.95E-06 | 0.0002 | 35 | 174743 |
|  |  | rs7160863 | *RP11-164H13.1* | 14 | 95791439 | 0.10 | A | G | 1.03 | 0.03 | 0.007 | 3.11E-06 | 0.0002 | 34 | 174743 |
| *Continued on next page* | | | | | | | | | | | | | | | |
|  |  |  |  |  |  |  |  |  |  |  |  |  |  |  |  |
| **Table S7. *Continued*** | | | | | | | | | | | | | | | |
| Fe | Secondary | rs201165754 | *ATAD2B* | 2 | 23921231 | 0.10 | A | C | 0.97 | -0.03 | 0.007 | 3.12E-06 | 0.0002 | 29 | 174743 |
|  |  | rs114081780 | *PTPRG* | 3 | 62130027 | 0.03 | G | A | 0.95 | -0.06 | 0.012 | 3.15E-06 | 0.0002 | 27 | 174743 |
|  |  | rs4367374 | *RP1-209A6.1* | 6 | 22767379 | 0.21 | C | A | 1.02 | 0.02 | 0.005 | 3.41E-06 | 0.0002 | 31 | 174743 |
|  |  | rs138682182 | *MICALL1* | 22 | 37908968 | 0.01 | A | G | 0.91 | -0.09 | 0.020 | 3.41E-06 | 0.0002 | 28 | 174743 |
|  |  | rs10159312 | *PCNX2* | 1 | 233019835 | 0.16 | T | C | 0.97 | -0.03 | 0.006 | 3.48E-06 | 0.0002 | 31 | 174743 |
|  |  | rs58658771 | *RP11-758N13.1* | 15 | 32709533 | 0.17 | A | T | 1.02 | 0.02 | 0.005 | 3.65E-06 | 0.0002 | 29 | 174743 |
|  |  | rs740516 | *ABCA6* | 17 | 69086821 | 0.15 | G | C | 0.97 | -0.03 | 0.006 | 3.94E-06 | 0.0002 | 30 | 174743 |
|  |  | rs77034634 | *NMU* | 4 | 54492079 | 0.06 | T | C | 1.04 | 0.04 | 0.008 | 4.13E-06 | 0.0002 | 27 | 174743 |
|  |  | rs563076 | *NEO1* | 15 | 73307469 | 0.38 | C | A | 1.02 | 0.02 | 0.004 | 4.16E-06 | 0.0002 | 29 | 174743 |
|  |  | rs61875518 | *BICC1* | 10 | 58469204 | 0.21 | A | G | 1.02 | 0.02 | 0.005 | 4.26E-06 | 0.0002 | 29 | 174743 |
|  |  | rs17019886 | *VIT* | 2 | 36820598 | 0.03 | G | T | 1.05 | 0.05 | 0.011 | 4.39E-06 | 0.0002 | 29 | 174743 |
|  |  | rs190262259 | *MIR4435-2HG* | 2 | 111362779 | 0.24 | A | G | 1.02 | 0.02 | 0.005 | 4.40E-06 | 0.0002 | 33 | 174743 |
|  |  | rs140047638 | *RNA5SP32* | 13 | 71979335 | 0.01 | C | T | 1.11 | 0.10 | 0.022 | 4.64E-06 | 0.0001 | 19 | 174743 |
|  |  | rs114165349 | *ARID1A* | 1 | 26695422 | 0.01 | C | G | 1.07 | 0.07 | 0.016 | 4.83E-06 | 0.0001 | 24 | 174743 |
|  |  | rs541857570 | *ZDHHC20P2* | 6 | 31378983 | 0.05 | T | C | 1.05 | 0.05 | 0.011 | 4.94E-06 | 0.0002 | 39 | 174743 |
| Se | Main | rs921943 | *DMGDH* | 5 | 79020653 | 0.27 | T | C | 1.28 | 0.25 | 0.023 | 9.40E-28 | 0.0240 | 135 | 5477 |
| Se | Secondary  QIMR | rs4950779 | *PPP1R12B* | 1 | 202573074 | 0.04 | C | T | 1.96 | 0.67 | 0.132 | 3.16E-07 | 0.0346 | 93 | 2603 |
|  |  | rs10023369 | *DPP3P1* | 4 | 62930825 | 0.49 | A | G | 0.87 | -0.14 | 0.029 | 4.42E-07 | 0.0104 | 27 | 2603 |
|  |  | rs7700970 | *BHMT* | 5 | 79115501 | 0.73 | C | T | 0.77 | -0.27 | 0.037 | 7.17E-13 | 0.0275 | 74 | 2603 |
|  |  | rs11779526 | *MIR548H4* | 8 | 27053199 | 0.66 | A | T | 0.86 | -0.15 | 0.032 | 1.68E-06 | 0.0105 | 28 | 2603 |
|  |  | rs7163368 | *APH1B* | 15 | 63278922 | 0.22 | C | T | 1.17 | 0.16 | 0.034 | 4.00E-06 | 0.0085 | 22 | 2603 |
|  |  | rs3785832 | *TBX4* | 17 | 61461433 | 0.56 | C | T | 0.86 | -0.15 | 0.031 | 1.82E-06 | 0.0105 | 28 | 2603 |
| *Continued on next page* | | | | | | | | | | | | | | | |
|  |  |  |  |  |  |  |  |  |  |  |  |  |  |  |  |
|  | | | | | | | | | | | | | | | |
|  |  |  |  |  |  |  |  |  |  |  |  |  |  |  |  |
| **Table S7. *Continued*** | | | | | | | | | | | | | | | |
| Se | Secondary  ALSPAC | rs3770549 | *MREG* | 2 | 215995827 | 0.33 | T | A | 1.21 | 0.19 | 0.040 | 2.39E-06 | 0.0154 | 45 | 2874 |
|  |  | rs6823178 | *CEP135* | 4 | 56008076 | 0.57 | G | A | 1.13 | 0.13 | 0.026 | 2.32E-06 | 0.0077 | 22 | 2874 |
|  |  | rs921943 | *-* | 5 | 79020653 | 0.27 | T | C | 1.30 | 0.26 | 0.030 | 1.43E-18 | 0.0276 | 82 | 2874 |
|  |  | rs11948804 | *HOMER1* | 5 | 79507955 | 0.10 | T | C | 1.27 | 0.24 | 0.047 | 3.90E-07 | 0.0096 | 28 | 2874 |
|  |  | rs2631524 | *RP11-26L20.3* | 16 | 55183220 | 0.18 | G | A | 1.16 | 0.15 | 0.032 | 1.98E-06 | 0.0068 | 20 | 2874 |
|  |  | rs12951643 | *HOXB1* | 17 | 48504656 | 0.94 | G | A | 1.26 | 0.23 | 0.047 | 8.01E-07 | 0.0065 | 19 | 2874 |
|  |  | rs9609603 | *SYN3* | 22 | 32621883 | 0.63 | T | C | 0.88 | -0.12 | 0.027 | 4.00E-06 | 0.0071 | 20 | 2874 |
| Zn | Main | rs1532423 | *CA1* | 8 | 85356084 | 0.37 | A | G | 1.19 | 0.18 | 0.026 | 6.40E-12 | 0.0148 | 39 | 2603 |
|  |  | rs2120019 | *PPCDC* | 15 | 75041843 | 0.20 | C | T | 0.75 | -0.29 | 0.033 | 1.55E-18 | 0.0267 | 71 | 2603 |
|  | Secondary | rs4333127 | *C4orf50* | 4 | 5928306 | 0.91 | A | G | 1.24 | 0.22 | 0.047 | 3.00E-06 | 0.0074 | 19 | 2603 |
|  |  | rs11763353 | *MEOX2* | 7 | 15591246 | 0.80 | A | G | 1.21 | 0.19 | 0.039 | 6.90E-07 | 0.0120 | 32 | 2603 |
|  |  | rs11232535 | *RP11-170L9.1* | 11 | 81217766 | 0.05 | C | T | 1.38 | 0.33 | 0.065 | 6.73E-07 | 0.0096 | 25 | 2603 |
|  |  | rs7148590 | *CHURC1-FNTB* | 14 | 65006478 | 0.56 | A | G | 0.87 | -0.14 | 0.026 | 1.37E-07 | 0.0097 | 25 | 2603 |
|  |  | rs10484100 | *RP11-26L16.1* | 14 | 86350752 | 0.92 | A | G | 1.23 | 0.21 | 0.045 | 3.30E-06 | 0.0065 | 17 | 2603 |
| Beta carotene | Main | rs6564851 | *BCMO1* | 16 | 81230992 | 0.50 | G | T | 1.16 | 0.15 | 0.015 | 1.60E-24 | 0.0111 | 44 | 3881 |
| Vitamin B12 | Main | rs602662 | *FUT2* | 19 | 48703728 | 0.60 | A | G | 1.17 | 0.16 | 0.006 | 2.40E-139 | 0.0123 | 569 | 45575 |
|  |  | rs34324219 | *TCN1* | 11 | 59855905 | 0.88 | C | A | 1.23 | 0.21 | 0.009 | 1.10E-111 | 0.0092 | 425 | 45575 |
|  |  | rs1801222 | *CUBN* | 10 | 17114152 | 0.59 | G | A | 1.12 | 0.11 | 0.008 | 2.30E-42 | 0.0058 | 268 | 45575 |
|  |  | rs2336573 | *CD320* | 19 | 8302825 | 0.03 | T | C | 1.38 | 0.32 | 0.020 | 8.40E-59 | 0.0062 | 282 | 45575 |
|  |  | rs1131603 | *TCN2* | 22 | 30622988 | 0.06 | C | T | 1.19 | 0.17 | 0.018 | 1.10E-21 | 0.0030 | 137 | 45575 |
|  |  | rs41281112 | *CLYBL* | 13 | 99866380 | 0.95 | C | T | 1.19 | 0.17 | 0.014 | 8.90E-35 | 0.0028 | 130 | 45575 |
|  |  | rs1141321 | *MUT* | 6 | 49444720 | 0.63 | C | T | 1.06 | 0.06 | 0.006 | 3.60E-26 | 0.0017 | 79 | 45575 |
|  |  | rs3742801 | *ABCD4* | 14 | 74292303 | 0.29 | T | C | 1.05 | 0.05 | 0.006 | 1.70E-13 | 0.0008 | 38 | 45575 |
|  |  | rs2270655 | *MMAA* | 4 | 145655266 | 0.94 | G | C | 1.07 | 0.07 | 0.009 | 2.20E-13 | 0.0005 | 22 | 45575 |
|  |  | rs12272669 | *SNRPCP14* | 11 | 71681564 | 0.00 | A | G | 1.67 | 0.51 | 0.088 | 3.00E-09 | 0.0011 | 52 | 45575 |
| *Continued on next page* | | | | | | | | | | | | | | | |
|  |  |  |  |  |  |  |  |  |  |  |  |  |  |  |  |
| **Table S7. *Continued*** | | | | | | | | | | | | | | | |
| Vitamin B12 | Secondary | rs62515066 | *RNU6-1213P* | 8 | 80435848 | 0.03 | G | A | 1.13 | 0.12 | 0.024 | 5.40E-07 | 0.0007 | 33 | 45575 |
| Vitamin C | Main | rs6693447 | *RER1* | 1 | 2398751 | 0.55 | T | G | 1.04 | 0.04 | 0.006 | 6.25E-10 | 0.0008 | 39 | 52018 |
|  |  | rs7740812 | *GSTA5* | 6 | 52860989 | 0.59 | G | A | 1.04 | 0.04 | 0.006 | 1.88E-09 | 0.0007 | 36 | 52018 |
|  |  | rs13028225 | *SLC23A3* | 2 | 219166533 | 0.86 | T | C | 1.11 | 0.10 | 0.009 | 2.38E-30 | 0.0026 | 133 | 52018 |
|  |  | rs33972313 | *SLC23A1* | 5 | 139379813 | 0.97 | C | T | 1.43 | 0.36 | 0.018 | 4.61E-90 | 0.0080 | 421 | 52018 |
|  |  | rs10051765 | *RGS14* | 5 | 177372991 | 0.34 | C | T | 1.04 | 0.04 | 0.007 | 3.64E-09 | 0.0007 | 36 | 52018 |
|  |  | rs174547 | *FADS1* | 11 | 61803311 | 0.33 | C | T | 1.04 | 0.04 | 0.007 | 3.84E-08 | 0.0006 | 30 | 52018 |
|  |  | rs117885456 | *SNRPF* | 12 | 95855333 | 0.09 | A | G | 1.08 | 0.08 | 0.012 | 1.70E-11 | 0.0010 | 50 | 52018 |
|  |  | rs2559850 | *CHPT1* | 12 | 101699681 | 0.60 | A | G | 1.06 | 0.06 | 0.006 | 6.30E-20 | 0.0016 | 84 | 52018 |
|  |  | rs10136000 | *AKT1* | 14 | 104787244 | 0.28 | A | G | 1.04 | 0.04 | 0.007 | 1.33E-08 | 0.0006 | 34 | 52018 |
|  |  | rs56738967 | *LINC01229* | 16 | 79706644 | 0.32 | C | G | 1.04 | 0.04 | 0.007 | 7.62E-10 | 0.0007 | 38 | 52018 |
|  |  | rs9895661 | *BCAS3* | 17 | 61379228 | 0.82 | T | C | 1.07 | 0.06 | 0.008 | 1.05E-14 | 0.0012 | 62 | 52018 |
|  | Secondary | rs77962988 | *ALMS1P1* | 2 | 73658963 | 0.84 | T | C | 0.96 | -0.05 | 0.008 | 9.00E-08 | 0.0006 | 29 | 52018 |
|  |  | rs73850547 | *CD200R1L* | 3 | 104587545 | 0.74 | A | G | 1.03 | 0.03 | 0.007 | 2.85E-06 | 0.0004 | 22 | 52018 |
|  |  | rs7640441 | *MRPL3* | 3 | 125399238 | 0.24 | A | C | 1.04 | 0.04 | 0.007 | 1.19E-06 | 0.0005 | 24 | 52018 |
|  |  | rs4867910 | *DOCK2* | 5 | 170014156 | 0.66 | T | C | 0.97 | -0.03 | 0.007 | 3.45E-06 | 0.0004 | 23 | 52018 |
|  |  | rs1165189 | *SLC17A3* | 6 | 25849551 | 0.75 | A | C | 0.96 | -0.04 | 0.007 | 1.18E-07 | 0.0005 | 28 | 52018 |
|  |  | rs4715312 | *GSTA2* | 6 | 52748683 | 0.50 | T | C | 1.03 | 0.03 | 0.006 | 9.07E-07 | 0.0005 | 25 | 52018 |
|  |  | rs868822 | *LINC01006* | 7 | 156460245 | 0.26 | T | G | 0.97 | -0.03 | 0.007 | 3.30E-06 | 0.0004 | 22 | 52018 |
|  |  | rs2941484 | *HNF4G* | 8 | 75566533 | 0.45 | T | C | 1.03 | 0.03 | 0.006 | 6.37E-08 | 0.0006 | 30 | 52018 |
|  |  | rs10758628 | *SLC1A1* | 9 | 4525855 | 0.46 | A | C | 1.03 | 0.03 | 0.006 | 1.28E-06 | 0.0005 | 24 | 52018 |
| Vitamin D | Main | rs10758628 | *SLC1A1* | 9 | 4525855 | 0.46 | A | C | 1.03 | 0.03 | 0.006 | 1.28E-06 | 0.0005 | 24 | 52018 |
|  |  | rs1011468 | *LINC01004* | 7 | 104973344 | 0.48 | A | G | 0.99 | -0.01 | 0.002 | 1.90E-12 | 0.0001 | 39 | 401460 |
|  |  | rs1047891 | *CPS1* | 2 | 210675783 | 0.32 | A | C | 0.99 | -0.01 | 0.002 | 1.00E-09 | 0.0001 | 29 | 401460 |
|  |  | rs10818769 | *RABGAP1* | 9 | 122957644 | 0.86 | G | C | 0.98 | -0.02 | 0.003 | 1.60E-09 | 0.0001 | 28 | 401460 |
|  |  | rs10859995 | *HAL* | 12 | 95981904 | 0.58 | C | T | 0.96 | -0.04 | 0.002 | 8.90E-81 | 0.0007 | 297 | 401460 |
| *Continued on next page* | | | | | | | | | | | | | | | |
|  |  |  |  |  |  |  |  |  |  |  |  |  |  |  |  |
| **Table S7. *Continued*** | | | | | | | | | | | | | | | |
| Vitamin D | Main | rs11127048 | *GCKR* | 2 | 27529596 | 0.62 | A | G | 1.02 | 0.02 | 0.002 | 3.00E-21 | 0.0002 | 76 | 401460 |
|  |  | rs111529171 | *DNAH11* | 7 | 21532314 | 0.22 | C | G | 0.99 | -0.02 | 0.002 | 3.10E-10 | 0.0001 | 31 | 401460 |
|  |  | rs112285002 | *SULT2A1* | 19 | 47871063 | 0.17 | T | C | 1.06 | 0.06 | 0.003 | 2.90E-115 | 0.0011 | 426 | 401460 |
|  |  | rs11264360 | *FDPS* | 1 | 155314795 | 0.24 | A | T | 1.02 | 0.02 | 0.002 | 6.90E-13 | 0.0001 | 43 | 401460 |
|  |  | rs1149605 | *RP11-21L23.4* | 11 | 76774172 | 0.17 | C | T | 1.02 | 0.02 | 0.003 | 1.10E-13 | 0.0001 | 46 | 401460 |
|  |  | rs117576073 | *CYP2R1* | 11 | 14891027 | 0.01 | T | G | 0.89 | -0.12 | 0.009 | 7.20E-38 | 0.0003 | 130 | 401460 |
|  |  | rs12123821 | *FLG-AS1* | 1 | 152206676 | 0.05 | T | C | 1.08 | 0.08 | 0.005 | 2.00E-58 | 0.0005 | 206 | 401460 |
|  |  | rs1229984 | *ADH1B* | 4 | 99318162 | 0.98 | C | T | 0.95 | -0.05 | 0.007 | 2.50E-13 | 0.0001 | 40 | 401460 |
|  |  | rs12317268 | *SLCO1B1* | 12 | 21199607 | 0.15 | G | A | 0.98 | -0.02 | 0.003 | 8.40E-12 | 0.0001 | 37 | 401460 |
|  |  | rs12803256 | *FLJ42102* | 11 | 71421822 | 0.78 | G | A | 1.11 | 0.10 | 0.002 | 1.00E-200 | 0.0035 | 1420 | 401460 |
|  |  | rs145432346 | *AC112518.3* | 4 | 71709300 | 0.83 | C | T | 1.11 | 0.11 | 0.003 | 1.00E-200 | 0.0034 | 1351 | 401460 |
|  |  | rs17765311 | *AC007950.2* | 15 | 63497753 | 0.34 | C | A | 0.98 | -0.02 | 0.002 | 1.60E-14 | 0.0001 | 46 | 401460 |
|  |  | rs1800588 | *LIPC* | 15 | 58431476 | 0.22 | T | C | 0.97 | -0.03 | 0.002 | 1.40E-35 | 0.0003 | 122 | 401460 |
|  |  | rs1800775 | *CETP* | 16 | 56961324 | 0.49 | A | C | 0.98 | -0.02 | 0.002 | 1.70E-17 | 0.0001 | 58 | 401460 |
|  |  | rs1972994 | *CADM2* | 3 | 85581992 | 0.65 | T | A | 0.98 | -0.02 | 0.002 | 3.00E-16 | 0.0001 | 53 | 401460 |
|  |  | rs2011425 | *UGT1A4* | 2 | 233718962 | 0.08 | G | T | 0.96 | -0.05 | 0.004 | 3.80E-32 | 0.0003 | 118 | 401460 |
|  |  | rs2037511 | *SERPINB11* | 18 | 63698973 | 0.17 | A | G | 1.02 | 0.02 | 0.003 | 2.90E-09 | 0.0001 | 32 | 401460 |
|  |  | rs2074735 | *PLA2G3* | 22 | 31139886 | 0.06 | C | G | 1.03 | 0.03 | 0.004 | 2.60E-11 | 0.0001 | 38 | 401460 |
|  |  | rs222026 | *GC* | 4 | 71778043 | 0.87 | T | A | 0.95 | -0.05 | 0.003 | 6.40E-62 | 0.0006 | 232 | 401460 |
|  |  | rs2229742 | *NRIP1* | 21 | 14966851 | 0.10 | C | G | 0.98 | -0.03 | 0.003 | 2.00E-13 | 0.0001 | 46 | 401460 |
|  |  | rs2847500 | *POU2F3* | 11 | 120243712 | 0.12 | A | G | 0.98 | -0.02 | 0.003 | 9.00E-14 | 0.0001 | 42 | 401460 |
|  |  | rs2909218 | *RP11-120M18.2* | 17 | 68468405 | 0.80 | T | C | 1.02 | 0.02 | 0.003 | 3.50E-11 | 0.0001 | 38 | 401460 |
|  |  | rs2934744 | *DOCK7* | 1 | 62582374 | 0.64 | A | C | 0.98 | -0.02 | 0.002 | 1.20E-25 | 0.0002 | 89 | 401460 |
|  |  | rs34726834 | *EBF2* | 8 | 26032090 | 0.25 | T | C | 1.01 | 0.01 | 0.002 | 1.60E-08 | 0.0001 | 26 | 401460 |
| *Continued on next page* | | | | | | | | | | | | | | | |
|  |  |  |  |  |  |  |  |  |  |  |  |  |  |  |  |
| **Table S7. *Continued*** | | | | | | | | | | | | | | | |
| Vitamin D | Main | rs3750296 | *PADI1* | 1 | 17233161 | 0.34 | C | G | 0.98 | -0.02 | 0.002 | 1.00E-22 | 0.0002 | 80 | 401460 |
|  |  | rs3814995 | *NPHS1* | 19 | 35851310 | 0.31 | T | C | 0.99 | -0.01 | 0.002 | 7.00E-11 | 0.0001 | 34 | 401460 |
|  |  | rs3822868 | *MED23* | 6 | 131613846 | 0.84 | G | A | 1.02 | 0.02 | 0.003 | 8.70E-16 | 0.0001 | 54 | 401460 |
|  |  | rs532436 | *ABO* | 9 | 133274414 | 0.18 | A | G | 0.99 | -0.02 | 0.003 | 1.70E-08 | 0.0001 | 27 | 401460 |
|  |  | rs577185477 | *PSMA1* | 11 | 14591017 | 0.01 | C | T | 0.69 | -0.38 | 0.010 | 1.00E-200 | 0.0034 | 1358 | 401460 |
|  |  | rs58073039 | *HSD17B11* | 4 | 87366211 | 0.30 | G | A | 0.99 | -0.02 | 0.002 | 6.70E-11 | 0.0001 | 38 | 401460 |
|  |  | rs58542926 | *TM6SF2* | 19 | 19268740 | 0.08 | T | C | 1.03 | 0.03 | 0.004 | 5.20E-19 | 0.0002 | 61 | 401460 |
|  |  | rs6127099 | *RP13-379L11.3* | 20 | 54114863 | 0.28 | T | A | 0.97 | -0.03 | 0.002 | 2.20E-47 | 0.0005 | 186 | 401460 |
|  |  | rs62007299 | *PEAK1* | 15 | 77419377 | 0.71 | A | G | 0.99 | -0.01 | 0.002 | 6.30E-11 | 0.0001 | 32 | 401460 |
|  |  | rs6438900 | *MRPL3* | 3 | 125429443 | 0.26 | G | C | 1.01 | 0.01 | 0.002 | 3.60E-10 | 0.0001 | 30 | 401460 |
|  |  | rs6698680 | *RER1* | 1 | 2398222 | 0.47 | G | A | 0.99 | -0.01 | 0.002 | 8.80E-09 | 0.0001 | 24 | 401460 |
|  |  | rs6724965 | *NPAS2* | 2 | 100823689 | 0.17 | G | A | 0.99 | -0.02 | 0.003 | 1.20E-08 | 0.0001 | 26 | 401460 |
|  |  | rs71383766 | *FBXL19* | 16 | 30918912 | 0.43 | T | C | 1.01 | 0.01 | 0.002 | 2.50E-10 | 0.0001 | 38 | 401460 |
|  |  | rs73015021 | *LDLR* | 19 | 11082239 | 0.12 | G | A | 1.02 | 0.02 | 0.003 | 2.20E-15 | 0.0001 | 49 | 401460 |
|  |  | rs7519574 | *RP4-657M3.2* | 1 | 34260951 | 0.18 | A | G | 1.02 | 0.02 | 0.003 | 2.20E-09 | 0.0001 | 31 | 401460 |
|  |  | rs7528419 | *CELSR2* | 1 | 109274570 | 0.23 | G | A | 1.02 | 0.02 | 0.002 | 2.00E-16 | 0.0001 | 51 | 401460 |
|  |  | rs7569755 | *HTR5BP* | 2 | 117890685 | 0.29 | A | G | 1.01 | 0.01 | 0.002 | 7.20E-11 | 0.0001 | 33 | 401460 |
|  |  | rs7650253 | *RHOA* | 3 | 49393727 | 0.69 | A | T | 1.02 | 0.02 | 0.002 | 5.20E-11 | 0.0001 | 39 | 401460 |
|  |  | rs7718395 | *TNFAIP8* | 5 | 119316879 | 0.32 | G | C | 1.01 | 0.01 | 0.002 | 1.20E-08 | 0.0001 | 25 | 401460 |
|  |  | rs77924615 | *PDILT* | 16 | 20381010 | 0.20 | A | G | 0.99 | -0.02 | 0.003 | 3.20E-09 | 0.0001 | 29 | 401460 |
|  |  | rs7828742 | *LINC00536* | 8 | 115948504 | 0.60 | G | A | 0.98 | -0.02 | 0.002 | 1.20E-28 | 0.0002 | 93 | 401460 |
|  |  | rs78649910 | *DOK7* | 4 | 3480486 | 0.11 | A | T | 0.98 | -0.02 | 0.003 | 8.40E-09 | 0.0001 | 28 | 401460 |
|  |  | rs8018720 | *SEC23A* | 14 | 39086981 | 0.82 | C | G | 0.97 | -0.03 | 0.003 | 6.50E-33 | 0.0003 | 105 | 401460 |
|  |  | rs804280 | *GATA4* | 8 | 11755189 | 0.58 | A | C | 1.01 | 0.01 | 0.002 | 1.40E-12 | 0.0001 | 38 | 401460 |
| *Continued on next page* | | | | | | | | | | | | | | | |
|  |  |  |  |  |  |  |  |  |  |  |  |  |  |  |  |
| **Table S7. *Continued*** | | | | | | | | | | | | | | | |
| Vitamin D | Main | rs8063706 | *BCAR4* | 16 | 11815695 | 0.27 | T | A | 1.01 | 0.01 | 0.002 | 7.50E-09 | 0.0001 | 31 | 401460 |
|  |  | rs8091117 | *DSG1* | 18 | 31339831 | 0.07 | A | C | 0.98 | -0.02 | 0.004 | 3.40E-09 | 0.0001 | 28 | 401460 |
|  |  | rs867772 | *MARC1* | 1 | 220799001 | 0.68 | G | A | 0.99 | -0.01 | 0.002 | 1.60E-11 | 0.0001 | 34 | 401460 |
|  |  | rs960596 | *SCUBE1* | 22 | 40997516 | 0.34 | T | C | 1.01 | 0.01 | 0.002 | 7.50E-09 | 0.0001 | 26 | 401460 |
|  |  | rs964184 | *ZPR1* | 11 | 116778201 | 0.87 | C | G | 1.04 | 0.04 | 0.003 | 2.50E-43 | 0.0004 | 154 | 401460 |
|  |  | rs9668081 | *FAM166AP9* | 12 | 38209109 | 0.47 | T | C | 1.01 | 0.01 | 0.002 | 1.30E-08 | 0.0001 | 29 | 401460 |
|  | Secondary | rs10500209 | *ZNF439* | 19 | 11868349 | 0.28 | C | T | 0.99 | -0.01 | 0.002 | 2.00E-09 | 0.0001 | 32 | 401460 |
|  |  | rs1065853 | *APOC1* | 19 | 44909976 | 0.08 | T | G | 1.03 | 0.03 | 0.004 | 2.30E-15 | 0.0001 | 47 | 401460 |
|  |  | rs10832218 | *SPON1* | 11 | 14159628 | 0.18 | C | T | 0.96 | -0.04 | 0.003 | 1.50E-29 | 0.0004 | 154 | 401460 |
|  |  | rs10832289 | *PDE3B* | 11 | 14647950 | 0.41 | T | A | 0.94 | -0.07 | 0.002 | 1.00E-200 | 0.0021 | 847 | 401460 |
|  |  | rs12997242 | *TDRD15* | 2 | 21158305 | 0.44 | A | G | 0.99 | -0.01 | 0.002 | 1.00E-09 | 0.0001 | 28 | 401460 |
|  |  | rs157595 | *APOC1* | 19 | 44922203 | 0.62 | G | A | 0.99 | -0.02 | 0.002 | 2.30E-12 | 0.0001 | 43 | 401460 |
|  |  | rs1858889 | *COG5* | 7 | 107477002 | 0.50 | C | A | 1.01 | 0.01 | 0.002 | 1.60E-09 | 0.0001 | 29 | 401460 |
|  |  | rs261291 | *ALDH1A2* | 15 | 58387979 | 0.36 | C | T | 0.98 | -0.02 | 0.002 | 3.60E-30 | 0.0003 | 106 | 401460 |
|  |  | rs2762942 | *CYP24A1* | 20 | 54172386 | 0.95 | A | G | 1.05 | 0.05 | 0.004 | 1.80E-30 | 0.0003 | 111 | 401460 |
|  |  | rs3768013 | *ARNT* | 1 | 150842935 | 0.37 | A | G | 0.99 | -0.01 | 0.002 | 4.60E-11 | 0.0001 | 37 | 401460 |
|  |  | rs523583 | *TMEM151A* | 11 | 66302675 | 0.47 | C | A | 1.01 | 0.01 | 0.002 | 2.30E-09 | 0.0001 | 29 | 401460 |
|  |  | rs56044892 | *FOXO6* | 1 | 41364414 | 0.21 | T | C | 1.01 | 0.01 | 0.003 | 5.80E-08 | 0.0001 | 23 | 401460 |
|  |  | rs57631352 | *STAP2* | 19 | 4338176 | 0.30 | G | A | 0.99 | -0.01 | 0.002 | 4.40E-08 | 0.0001 | 28 | 401460 |
|  |  | rs6123359 | *RP13-379L11.3* | 20 | 54098167 | 0.11 | G | A | 1.03 | 0.03 | 0.003 | 1.80E-21 | 0.0002 | 73 | 401460 |
|  |  | rs7699711 | *UGT2B7* | 4 | 69081878 | 0.72 | T | C | 1.01 | 0.01 | 0.002 | 1.80E-07 | 0.0001 | 23 | 401460 |
|  |  | rs8103262 | *ZNF808* | 19 | 52562561 | 0.31 | C | T | 1.01 | 0.01 | 0.002 | 4.00E-07 | 0.0001 | 25 | 401460 |
| ^a^ SNPs with *r^2^* < 0.001 within 10,000 kb windows and *P* ≤ 5E-08 were used for the main analyses, while SNPs with *r^2^* < 0.01 within 10,000 kb and *P* ≤ 5E-06 were used for the secondary analyses.  ^b^ Gene mapping based on PhenoScanner V2  ^c^ Position based on GRCh38.  ^d^ The odds ratios (OR) correspond to a 1 standard deviation increase in the concentration of the micronutrient.  ^e^ The beta-coefficient from Jäger et al. was converted from computed Z-scores.  Abbreviations: BP, base position; chromosome position; CHR, chromosome; Cu, Copper; EA, effect allele; EAF, effect allele frequency; Fe, Iron; N, sample size; OA, other allele; OR, odds ratio; R^2^, Proportion of the explained variance; Se, Selenium; StdErr, standard error; Zn, Zinc. | | | | | | | | | | | | | | | |

| **Table S8.** Main mendelian randomization analyses of micronutrients as risk factors on the risk of gastrointestinal infections | | | | | | | | | | | | | | | | |
| --- | --- | --- | --- | --- | --- | --- | --- | --- | --- | --- | --- | --- | --- | --- | --- | --- |
|  |  |  | **Meta-analysis** | | | |  | **UK Biobank** | | | |  | **FinnGen R6** | | | |
| **Exposure^a^** | **Method** |  | **Number**  **of SNPs** | **OR^b^**  **(95% CI)** | ***P* value** | **Cochran's**  **Q** |  | **Number**  **of SNPs** | **OR^b^**  **(95% CI)** | ***P* value** | **Cochran's**  **Q** |  | **Number**  **of SNPs** | **OR^b^**  **(95% CI)** | ***P* value** | **Cochran's**  **Q** |
| Cu | IVW |  | 2 | 0.91  (0.87 - 0.97) | 1.38E-03 | 5.99E-01 |  | 2 | 0.89  (0.80 - 0.98) | 1.67E-02 | 6.92E-01 |  | 2 | 0.93  (0.87 - 0.99) | 1.98E-02 | 7.34E-01 |
| Fe^c^ | MR Egger |  | 12 | 1.00  (0.91 - 1.11) | 9.27E-01 | 8.52E-01 |  | 12 | 1.16  (0.98 - 1.37) | 1.20E-01 | 9.67E-01 |  | 11 | 0.93  (0.82 - 1.05) | 2.74E-01 | 4.59E-01 |
|  | Weighted median |  | 12 | 1.01  (0.93 - 1.10) | 8.29E-01 |  |  | 12 | 1.08  (0.94 - 1.25) | 2.78E-01 |  |  | 11 | 0.98  (0.88 - 1.09) | 7.09E-01 |  |
|  | IVW |  | 12 | 1.00  (0.93 - 1.07) | 9.89E-01 | 9.01E-01 |  | 12 | 1.03  (0.92 - 1.16) | 5.73E-01 | 8.15E-01 |  | 11 | 0.98  (0.90 - 1.07) | 6.74E-01 | 4.22E-01 |
|  | Simple mode |  | 12 | 0.98  (0.84 - 1.15) | 8.36E-01 |  |  | 12 | 0.95  (0.72 - 1.25) | 7.07E-01 |  |  | 11 | 0.99  (0.82 - 1.19) | 9.09E-01 |  |
|  | Weighted mode |  | 12 | 1.01  (0.93 - 1.09) | 8.29E-01 |  |  | 12 | 1.08  (0.95 - 1.22) | 2.75E-01 |  |  | 11 | 0.98  (0.89 - 1.07) | 6.42E-01 |  |
| Se | Wald ratio |  | 1 | 0.92  (0.85 - 0.99) | 2.39E-02 |  |  | 1 | 0.94  (0.83 - 1.08) | 4.10E-01 |  |  | 1 | 0.91  (0.83 - 0.99) | 2.63E-02 |  |
| Zn | IVW |  | 2 | 0.99  (0.94 - 1.06) | 8.65E-01 | 2.76E-01 |  | 2 | 0.92  (0.80 - 1.06) | 2.39E-01 | 1.73E-01 |  | 2 | 1.03  (0.96 - 1.10) | 3.99E-01 | 6.09E-01 |
| Beta carotene | Wald ratio |  | 1 | 0.93  (0.84 - 1.04) | 2.29E-01 |  |  | 1 | 0.96  (0.79 - 1.17) | 6.60E-01 |  |  | 1 | 0.92  (0.81 - 1.05) | 2.35E-01 |  |
| Vitamin B12 | MR Egger |  | 9 | 0.89  (0.70 - 1.12) | 3.44E-01 | 3.38E-04 |  | 9 | 0.84  (0.68 - 1.04) | 1.49E-01 | 5.85E-01 |  | 9 | 0.91  (0.69 - 1.20) | 5.27E-01 | 2.34E-04 |
|  | Weighted median |  | 9 | 0.97  (0.87 - 1.07) | 5.36E-01 |  |  | 9 | 1.04  (0.91 - 1.19) | 5.84E-01 |  |  | 9 | 0.93  (0.82 - 1.05) | 2.25E-01 |  |
|  | IVW |  | 9 | 0.97  (0.87 - 1.08) | 6.01E-01 | 2.19E-04 |  | 9 | 1.06  (0.94 - 1.21) | 3.31E-01 | 1.52E-01 |  | 9 | 0.93  (0.83 - 1.06) | 2.80E-01 | 4.70E-04 |
|  | Simple mode |  | 9 | 0.97  (0.79 - 1.19) | 7.74E-01 |  |  | 9 | 1.13  (0.94 - 1.35) | 2.21E-01 |  |  | 9 | 1.01  (0.83 - 1.23) | 9.04E-01 |  |
|  | Weighted mode |  | 9 | 1.09  (0.83 - 1.43) | 5.50E-01 |  |  | 9 | 1.03  (0.90 - 1.17) | 7.01E-01 |  |  | 9 | 1.08  (0.85 - 1.36) | 5.54E-01 |  |
| *Continued on next page* | | | | | | | | | | | | | | | | |
|  |  |  |  |  |  |  |  |  |  |  |  |  |  |  |  |  |
|  |  |  |  |  |  |  |  |  |  |  |  |  |  |  |  |  |
| **Table S8. *Continued*** | | | | | | | | | | | | | | | | |
| Vitamin C^d^ | MR Egger |  | 10 | 0.87  (0.73 - 1.03) | 1.44E-01 | 3.03E-01 |  | 10 | 0.80  (0.63 - 1.02) | 1.13E-01 | 6.80E-01 |  | 9 | 0.90  (0.74 - 1.10) | 3.29E-01 | 4.49E-01 |
|  | Weighted median |  | 10 | 0.91  (0.80 - 1.03) | 1.51E-01 |  |  | 10 | 0.95  (0.78 - 1.16) | 6.33E-01 |  |  | 9 | 0.89  (0.77 - 1.04) | 1.44E-01 |  |
|  | IVW |  | 10 | 0.96  (0.86 - 1.07) | 4.29E-01 | 2.23E-01 |  | 10 | 1.02  (0.85 - 1.22) | 8.37E-01 | 2.14E-01 |  | 9 | 0.92  (0.82 - 1.04) | 1.68E-01 | 5.49E-01 |
|  | Simple mode |  | 10 | 1.01  (0.82 - 1.23) | 9.37E-01 |  |  | 10 | 1.04  (0.72 - 1.49) | 8.55E-01 |  |  | 9 | 0.86  (0.69 - 1.08) | 2.41E-01 |  |
|  | Weighted mode |  | 10 | 0.89  (0.78 - 1.02) | 1.23E-01 |  |  | 10 | 0.90  (0.72 - 1.11) | 3.55E-01 |  |  | 9 | 0.88  (0.75 - 1.04) | 1.73E-01 |  |
| Vitamin D^e^ | MR Egger |  | 69 | 1.03  (0.90 - 1.17) | 7.02E-01 | 6.06E-01 |  | 64 | 0.97  (0.75 - 1.26) | 8.46E-01 | 1.28E-01 |  | 64 | 1.05  (0.90 - 1.22) | 5.49E-01 | 6.06E-01 |
|  | Weighted median |  | 69 | 1.08  (0.95 - 1.24) | 2.33E-01 |  |  | 64 | 1.02  (0.77 - 1.36) | 8.71E-01 |  |  | 64 | 1.05  (0.91 - 1.22) | 4.75E-01 |  |
|  | IVW |  | 69 | 1.11  (1.02 - 1.21) | 2.00E-02 | 5.56E-01 |  | 64 | 1.08  (0.90 - 1.28) | 4.04E-01 | 1.26E-01 |  | 64 | 1.12  (1.01 - 1.24) | 2.48E-02 | 5.90E-01 |
|  | Simple mode |  | 69 | 1.07  (0.83 - 1.39) | 5.84E-01 |  |  | 64 | 1.26  (0.71 - 2.25) | 4.30E-01 |  |  | 64 | 1.28  (0.95 - 1.73) | 1.13E-01 |  |
|  | Weighted mode |  | 69 | 1.05  (0.93 - 1.19) | 4.24E-01 |  |  | 64 | 1.08  (0.85 - 1.38) | 5.36E-01 |  |  | 64 | 1.05  (0.91 - 1.20) | 4.98E-01 |  |
| ^a^ Only independent SNPs (*r^2^* < 0.001 within 10,000 kb windows), strongly associated (*P* ≤ 5E-08) were used as genetic instruments for the exposure.  ^b^ The odds ratios (OR) correspond to a 1 standard deviation increase in the concentration of the micronutrient.  ^c^ For Fe, 12 SNPs were available in UK Biobank, while 11 SNPs was available in FinnGen R6, resulting in 12 SNPs for the meta-analysis.  ^d^ For vitamin C, 10 SNPs were available in UK Biobank, and 9 SNP was available in FinnGen R6, resulting 10 SNP for the meta-analysis.  ^e^ For vitamin D, 64 SNPs were available in UK Biobank, and 64 SNP was available in FinnGen R6, resulting 69 SNP for the meta-analysis.  Abbreviations: Cu: Copper, Fe: Iron, IVW: inverse-variance weighted, Se: Selenium, Zn: Zinc | | | | | | | | | | | | | | | |  |

| **Table S9.** Main mendelian randomization analyses of micronutrients as risk factors on the risk of pneumonia. | | | | | | | | | | | | | | | | |
| --- | --- | --- | --- | --- | --- | --- | --- | --- | --- | --- | --- | --- | --- | --- | --- | --- |
|  |  |  | **Meta-analysis** | | | |  | **UK Biobank** | | | |  | **FinnGen R6** | | | |
| **Exposure** | **Method** |  | **Number**  **of SNPs** | **OR^b^**  **(95% CI)** | ***P* value** | **Cochran's**  **Q** |  | **Number**  **of SNPs** | **OR^b^**  **(95% CI)** | ***P* value** | **Cochran's**  **Q** |  | **Number**  **of SNPs** | **OR^b^**  **(95% CI)** | ***P* value** | **Cochran's**  **Q** |
| Cu | IVW |  | 2 | 0.95  (0.88 - 1.02) | 1.77E-01 | 7.56E-01 |  | 2 | 0.94  (0.83 - 1.05) | 2.78E-01 | 3.24E-01 |  | 2 | 0.96  (0.86 - 1.06) | 3.71E-01 | 7.04E-01 |
| Fe^c^ | MR Egger |  | 12 | 1.04  (0.83 - 1.30) | 7.48E-01 | 6.67E-03 |  | 12 | 1.09  (0.83 - 1.43) | 5.38E-01 | 6.06E-02 |  | 11 | 0.99  (0.72 - 1.36) | 9.36E-01 | 6.58E-03 |
|  | Weighted median |  | 12 | 1.03  (0.89 - 1.18) | 7.19E-01 |  |  | 12 | 1.09  (0.90 - 1.31) | 3.83E-01 |  |  | 11 | 0.89  (0.75 - 1.06) | 2.00E-01 |  |
|  | IVW |  | 12 | 1.01  (0.87 - 1.18) | 8.74E-01 | 1.04E-02 |  | 12 | 1.06  (0.89 - 1.27) | 4.91E-01 | 8.65E-02 |  | 11 | 0.96  (0.78 - 1.19) | 7.30E-01 | 1.10E-02 |
|  | Simple mode |  | 12 | 0.83  (0.58 - 1.19) | 3.29E-01 |  |  | 12 | 0.87  (0.57 - 1.33) | 5.37E-01 |  |  | 11 | 0.59  (0.39 - 0.91) | 3.81E-02 |  |
|  | Weighted mode |  | 12 | 0.96  (0.83 - 1.11) | 5.96E-01 |  |  | 12 | 1.09  (0.93 - 1.28) | 3.30E-01 |  |  | 11 | 0.95  (0.80 - 1.12) | 5.55E-01 |  |
| Se | Wald ratio |  | 1 | 1.01  (0.91 - 1.12) | 8.60E-01 |  |  | 1 | 1.03  (0.88 - 1.19) | 7.48E-01 |  |  | 1 | 1.00  (0.87 - 1.14) | 9.64E-01 |  |
| Zn | IVW |  | 2 | 0.94  (0.87 - 1.02) | 1.55E-01 | 7.22E-01 |  | 2 | 1.03  (0.91 - 1.16) | 6.79E-01 | 7.54E-01 |  | 2 | 0.89  (0.80 - 0.98) | 2.40E-02 | 8.83E-01 |
| Beta carotene | Wald ratio |  | 1 | 1.10  (0.94 - 1.29) | 2.41E-01 |  |  | 1 | 1.08  (0.86 - 1.37) | 5.05E-01 |  |  | 1 | 1.11  (0.91 - 1.37) | 3.09E-01 |  |
| Vitamin B12 | MR Egger |  | 9 | 1.08  (0.88 - 1.32) | 5.12E-01 | 1.51E-01 |  | 9 | 1.04  (0.79 - 1.37) | 7.80E-01 | 2.52E-01 |  | 9 | 1.11  (0.86 - 1.42) | 4.58E-01 | 2.32E-01 |
|  | Weighted median |  | 9 | 1.10  (0.98 - 1.22) | 1.03E-01 |  |  | 9 | 1.10  (0.93 - 1.30) | 2.60E-01 |  |  | 9 | 1.06  (0.92 - 1.22) | 4.02E-01 |  |
|  | IVW |  | 9 | 1.09  (0.99 - 1.19) | 7.69E-02 | 2.16E-01 |  | 9 | 1.14  (1.00 - 1.31) | 4.94E-02 | 2.84E-01 |  | 9 | 1.05  (0.94 - 1.17) | 4.04E-01 | 2.96E-01 |
|  | Simple mode |  | 9 | 1.16  (1.00 - 1.35) | 8.85E-02 |  |  | 9 | 1.02  (0.83 - 1.27) | 8.33E-01 |  |  | 9 | 1.07  (0.86 - 1.35) | 5.56E-01 |  |
|  | Weighted mode |  | 9 | 1.11  (0.99 - 1.24) | 1.18E-01 |  |  | 9 | 1.11  (0.92 - 1.34) | 2.97E-01 |  |  | 9 | 1.05  (0.90 - 1.22) | 5.48E-01 |  |
| *Continued on next page* | | | | | | | | | | | | | | | | |
|  |  |  |  |  |  |  |  |  |  |  |  |  |  |  |  |  |
|  |  |  |  |  |  |  |  |  |  |  |  |  |  |  |  |  |
| **Table S9. *Continued*** | | | | | | | | | | | | | | | | |
| Vitamin C^d^ | MR Egger |  | 10 | 1.01  (0.73 - 1.38) | 9.71E-01 | 2.27E-02 |  | 10 | 0.97  (0.70 - 1.35) | 8.77E-01 | 2.25E-01 |  | 9 | 1.04  (0.71 - 1.54) | 8.38E-01 | 1.37E-01 |
|  | Weighted median |  | 10 | 1.00  (0.84 - 1.18) | 9.60E-01 |  |  | 10 | 0.97  (0.78 - 1.22) | 8.15E-01 |  |  | 9 | 1.04  (0.82 - 1.32) | 7.50E-01 |  |
|  | IVW |  | 10 | 1.02  (0.85 - 1.23) | 8.20E-01 | 3.70E-02 |  | 10 | 1.01  (0.83 - 1.23) | 8.96E-01 | 2.95E-01 |  | 9 | 1.03  (0.83 - 1.28) | 7.78E-01 | 1.99E-01 |
|  | Simple mode |  | 10 | 1.04  (0.80 - 1.36) | 7.61E-01 |  |  | 10 | 1.04  (0.68 - 1.58) | 8.69E-01 |  |  | 9 | 1.09  (0.74 - 1.62) | 6.62E-01 |  |
|  | Weighted mode |  | 10 | 1.01  (0.83 - 1.22) | 9.42E-01 |  |  | 10 | 0.98  (0.77 - 1.26) | 8.86E-01 |  |  | 9 | 1.05  (0.82 - 1.36) | 6.94E-01 |  |
| Vitamin D^e^ | MR Egger |  | 69 | 1.08  (0.88 - 1.33) | 4.85E-01 | 8.11E-02 |  | 64 | 1.00  (0.72 - 1.38) | 9.89E-01 | 3.42E-02 |  | 64 | 1.14  (0.90 - 1.44) | 2.94E-01 | 4.53E-01 |
|  | Weighted median |  | 69 | 1.12  (0.94 - 1.34) | 2.04E-01 |  |  | 64 | 1.07  (0.82 - 1.40) | 6.02E-01 |  |  | 64 | 1.18  (0.92 - 1.50) | 1.90E-01 |  |
|  | IVW |  | 69 | 1.05  (0.92 - 1.20) | 4.86E-01 | 9.27E-02 |  | 64 | 0.97  (0.78 - 1.20) | 7.91E-01 | 4.08E-02 |  | 64 | 1.11  (0.95 - 1.30) | 1.97E-01 | 4.86E-01 |
|  | Simple mode |  | 69 | 1.08  (0.75 - 1.55) | 6.89E-01 |  |  | 64 | 1.28  (0.72 - 2.25) | 4.02E-01 |  |  | 64 | 1.09  (0.65 - 1.84) | 7.48E-01 |  |
|  | Weighted mode |  | 69 | 1.09  (0.93 - 1.28) | 2.87E-01 |  |  | 64 | 1.05  (0.83 - 1.33) | 6.92E-01 |  |  | 64 | 1.09  (0.89 - 1.34) | 4.12E-01 |  |
| ^a^ Only independent SNPs (*r^2^* < 0.001 within 10,000 kb windows), strongly associated (*P* ≤ 5E-08) were used as genetic instruments for the exposure.  ^b^ The odds ratios (OR) correspond to a 1 standard deviation increase in the concentration of the micronutrient.  ^c^ For Fe, 12 SNPs were available in UK Biobank, while 11 SNPs was available in FinnGen R6, resulting in 12 SNPs for the meta-analysis.  ^d^ For vitamin C, 10 SNPs were available in UK Biobank, and 9 SNP was available in FinnGen R6, resulting 10 SNP for the meta-analysis.  ^e^ For vitamin D, 64 SNPs were available in UK Biobank, and 64 SNP was available in FinnGen R6, resulting 69 SNP for the meta-analysis.  Abbreviations: Cu: Copper, Fe: Iron, IVW: inverse-variance weighted, Se: Selenium, Zn: Zinc | | | | | | | | | | | | | | | | |

| **Table S10**. Main mendelian randomization analyses of micronutrients as risk factors on the risk of urinary tract infections. | | | | | | | | | | | | | | | | |
| --- | --- | --- | --- | --- | --- | --- | --- | --- | --- | --- | --- | --- | --- | --- | --- | --- |
|  |  |  |  | **Meta-analysis** | | |  | **UK Biobank** | | | |  | **FinnGen R6** | | | |
| **Exposure^a^** | **Method** | **Number**  **of SNPs** |  | **OR^b^**  **(95% CI)** | ***P* value** | **Cochran's**  **Q** |  | **Number**  **of SNPs** | **OR^b^**  **(95% CI)** | ***P* value** | **Cochran's Q** |  | **Number**  **of SNPs** | **OR^b^**  **(95% CI)** | ***P* value** | **Cochran's**  **Q** |
| Cu | IVW | 2 |  | 0.99  (0.94 - 1.05) | 7.86E-01 | 8.62E-01 |  | 2 | 1.03  (0.94 - 1.12) | 5.15E-01 | 7.24E-01 |  | 2 | 0.96  (0.89 - 1.04) | 3.27E-01 | 9.65E-01 |
| Fe^c^ | MR Egger | 12 |  | 1.04  (0.9 - 1.20) | 6.36E-01 | 5.09E-02 |  | 12 | 1.07  (0.91 - 1.25) | 4.19E-01 | 2.98E-01 |  | 11 | 0.99  (0.81 - 1.22) | 9.55E-01 | 5.85E-02 |
|  | Weighted median | 12 |  | 1.07  (0.98 - 1.17) | 1.45E-01 |  |  | 12 | 1.13  (0.99 - 1.29) | 6.41E-02 |  |  | 11 | 1.01  (0.89 - 1.14) | 9.03E-01 |  |
|  | IVW | 12 |  | 1.06  (0.96 - 1.16) | 2.57E-01 | 7.07E-02 |  | 12 | 1.12  (1.01 - 1.25) | 3.43E-02 | 3.18E-01 |  | 11 | 0.99  (0.86 - 1.13) | 8.31E-01 | 8.74E-02 |
|  | Simple mode | 12 |  | 1.01  (0.86 - 1.18) | 9.26E-01 |  |  | 12 | 1.15  (0.89 - 1.47) | 3.16E-01 |  |  | 11 | 0.99  (0.79 - 1.24) | 9.09E-01 |  |
|  | Weighted mode | 12 |  | 1.06  (0.98 - 1.15) | 1.87E-01 |  |  | 12 | 1.11  (0.99 - 1.25) | 9.46E-02 |  |  | 11 | 1.00  (0.89 - 1.14) | 9.60E-01 |  |
| Se | Wald ratio | 1 |  | 1.03  (0.95 - 1.11) | 4.53E-01 |  |  | 1 | 1.09  (0.98 - 1.22) | 1.16E-01 |  |  | 1 | 0.98  (0.88 - 1.08) | 6.44E-01 |  |
| Zn | IVW | 2 |  | 1.00  (0.95 - 1.06) | 9.07E-01 | 4.49E-01 |  | 2 | 1.08  (0.99 - 1.17) | 1.02E-01 | 6.21E-01 |  | 2 | 0.95  (0.88 - 1.02) | 1.68E-01 | 6.13E-01 |
| Beta carotene | Wald ratio | 1 |  | 1.01  (0.90 - 1.13) | 8.92E-01 |  |  | 1 | 0.89  (0.75 - 1.06) | 1.91E-01 |  |  | 1 | 1.12  (0.96 - 1.31) | 1.47E-01 |  |
| Vitamin B12 | MR Egger | 9 |  | 1.01  (0.86 - 1.18) | 9.27E-01 | 1.19E-01 |  | 9 | 1.07  (0.90 - 1.28) | 4.78E-01 | 5.52E-01 |  | 9 | 0.95  (0.77 - 1.18) | 6.50E-01 | 9.45E-02 |
|  | Weighted median | 9 |  | 1.03  (0.95 - 1.13) | 4.54E-01 |  |  | 9 | 0.95  (0.85 - 1.07) | 4.35E-01 |  |  | 9 | 1.01  (0.9 - 1.12) | 8.94E-01 |  |
|  | IVW | 9 |  | 1.00  (0.93 - 1.07) | 9.33E-01 | 1.75E-01 |  | 9 | 0.97  (0.88 - 1.05) | 4.38E-01 | 4.74E-01 |  | 9 | 1.02  (0.93 - 1.13) | 6.57E-01 | 1.04E-01 |
|  | Simple mode | 9 |  | 1.07  (0.93 - 1.23) | 3.91E-01 |  |  | 9 | 0.97  (0.80 - 1.18) | 7.86E-01 |  |  | 9 | 1.14  (0.97 - 1.34) | 1.63E-01 |  |
|  | Weighted mode | 9 |  | 1.07  (0.93 - 1.22) | 3.70E-01 |  |  | 9 | 0.95  (0.82 - 1.10) | 5.11E-01 |  |  | 9 | 0.95  (0.85 - 1.08) | 4.66E-01 |  |
| *Continued on next page* | | | | | | | | | | | | | | | | |
|  |  |  |  |  |  |  |  |  |  |  |  |  |  |  |  |  |
|  |  |  |  |  |  |  |  |  |  |  |  |  |  |  |  |  |
| **Table S10. *Continued*** | | | | | | | | | | | | | | | | |
| Vitamin C^d^ | MR Egger | 10 |  | 1.07  (0.91 - 1.26) | 4.44E-01 | 3.53E-01 |  | 10 | 1.08  (0.85 - 1.37) | 5.30E-01 | 2.19E-01 |  | 9 | 1.05  (0.83 - 1.33) | 6.83E-01 | 6.21E-01 |
|  | Weighted median | 10 |  | 0.99  (0.88 - 1.12) | 8.94E-01 |  |  | 10 | 1.01  (0.85 - 1.18) | 9.51E-01 |  |  | 9 | 1.04  (0.87 - 1.24) | 6.74E-01 |  |
|  | IVW | 10 |  | 0.97  (0.86 - 1.08) | 5.30E-01 | 2.37E-01 |  | 10 | 0.98  (0.84 - 1.14) | 7.77E-01 | 1.97E-01 |  | 9 | 0.95  (0.83 - 1.09) | 4.68E-01 | 5.94E-01 |
|  | Simple mode | 10 |  | 0.97  (0.79 - 1.21) | 8.18E-01 |  |  | 10 | 0.94  (0.71 - 1.24) | 6.70E-01 |  |  | 9 | 0.94  (0.70 - 1.26) | 7.05E-01 |  |
|  | Weighted mode | 10 |  | 1.02  (0.90 - 1.16) | 7.46E-01 |  |  | 10 | 1.01  (0.85 - 1.19) | 9.43E-01 |  |  | 9 | 1.02  (0.83 - 1.25) | 8.57E-01 |  |
| Vitamin D^e^ | MR Egger | 69 |  | 0.98  (0.84 - 1.15) | 8.42E-01 | 1.91E-02 |  | 64 | 1.06  (0.85 - 1.31) | 6.16E-01 | 1.92E-01 |  | 64 | 0.93  (0.77 - 1.12) | 4.37E-01 | 2.10E-01 |
|  | Weighted median | 69 |  | 0.98  (0.86 - 1.13) | 7.91E-01 |  |  | 64 | 1.00  (0.81 - 1.23) | 9.94E-01 |  |  | 64 | 0.95  (0.79 - 1.14) | 6.00E-01 |  |
|  | IVW | 69 |  | 1.00  (0.90 - 1.12) | 9.43E-01 | 2.27E-02 |  | 64 | 1.03  (0.90 - 1.19) | 6.52E-01 | 2.15E-01 |  | 64 | 0.98  (0.87 - 1.11) | 7.58E-01 | 2.19E-01 |
|  | Simple mode | 69 |  | 0.91  (0.64 - 1.3) | 6.14E-01 |  |  | 64 | 1.06  (0.68 - 1.65) | 7.88E-01 |  |  | 64 | 0.9  (0.60 - 1.34) | 6.04E-01 |  |
|  | Weighted mode | 69 |  | 0.96  (0.84 - 1.09) | 5.18E-01 |  |  | 64 | 1.03  (0.86 - 1.24) | 7.16E-01 |  |  | 64 | 0.93  (0.79 - 1.09) | 3.60E-01 |  |
| ^a^ Only independent SNPs (*r^2^* < 0.001 within 10,000 kb windows), strongly associated (*P* ≤ 5E-08) were used as genetic instruments for the exposure.  ^b^ The odds ratios (OR) correspond to a 1 standard deviation increase in the concentration of the micronutrient.  ^c^ For Fe, 12 SNPs were available in UK Biobank, while 11 SNPs was available in FinnGen R6, resulting in 12 SNPs for the meta-analysis.  ^d^ For vitamin C, 10 SNPs were available in UK Biobank, and 9 SNP was available in FinnGen R6, resulting 10 SNP for the meta-analysis.  ^e^ For vitamin D, 64 SNPs were available in UK Biobank, and 64 SNP was available in FinnGen R6, resulting 69 SNP for the meta-analysis.  Abbreviations: Cu: Copper, Fe: Iron, IVW: inverse-variance weighted, Se: Selenium, Zn: Zinc | | | | | | | | | | | | | | | | |

| **Table S11.** Secondary mendelian randomization analyses of micronutrients as risk factors on the risk of gastrointestinal infections, pneumonia and urinary tract infections suggestive-significant genetic instruments. | | | | | | | | | | | | | | |
| --- | --- | --- | --- | --- | --- | --- | --- | --- | --- | --- | --- | --- | --- | --- |
|  |  |  |  | **Gastrointestinal infections** | | |  | **Pneumonia** | | |  | **Urinary tract infections** | | |
| Exposure^a^ | Method | **Number of SNPs** |  | **OR^b^**  **(95% CI)** | ***P* value** | **Cochran's Q** |  | **OR^b^**  **(95% CI)** | ***P* value** | **Cochran's Q** |  | **OR^b^**  **(95% CI)** | ***P* value** | **Cochran's Q** |
| Cu Evans et al. | MR Egger | 6 |  | 1.01  (0.94 - 1.09) | 7.18E-01 | 2.28E-03 |  | 1.00  (0.95 - 1.06) | 9.96E-01 | 6.50E-01 |  | 1.00  (0.96 - 1.05) | 8.37E-01 | 4.47E-01 |
|  | Weighted median | 6 |  | 1.02  (0.98 - 1.06) | 3.66E-02 |  |  | 1.00  (0.96 - 1.05) | 9.46E-01 |  |  | 1.01  (0.97 - 1.05) | 5.41E-01 |  |
|  | IVW | 6 |  | 1.01  (0.96 - 1.06) | 7.45E-01 | 4.68E-03 |  | 0.99  (0.96 - 1.03) | 7.03E-01 | 7.61E-01 |  | 1.02  (0.99 - 1.05) | 2.49E-01 | 5.06E-01 |
|  | Simple mode | 6 |  | 1.05  (0.97 - 1.14) | 2.63E-01 |  |  | 0.97  (0.89 - 1.05) | 4.55E-01 |  |  | 1.00  (0.95 - 1.06) | 8.49E-01 |  |
|  | Weighted mode | 6 |  | 2.03  (0.99 - 1.14) | 2.63E-02 |  |  | 1.00  (0.96 - 1.05) | 9.09E-01 |  |  | 1.00  (0.98 - 1.04) | 6.12E-01 |  |
|  | RAPS | 6 |  | 1.02  (0.98 - 1.06) | 3.32E-01 |  |  | 0.99  (0.95 - 1.03) | 7.09E-01 |  |  | 1.02  (0.97 - 1.07) | 4.28E-01 |  |
| Cu Jäger et al.^c^ | MR Egger | 7 |  | 1.07  (0.93 - 1.23) | 3.99E-01 | 7.16E-01 |  | 0.94  (0.77 - 1.15) | 5.82E-01 | 7.29E-01 |  | 1.01  (0.88 - 1.17) | 8.72E-01 | 5.18E-01 |
|  | Weighted median | 7 |  | 0.98  (0.92 - 1.04) | 4.42E-01 |  |  | 0.96  (0.88 - 1.04) | 3.19E-01 |  |  | 1.02  (0.96 - 1.08) | 5.98E-01 |  |
|  | IVW | 7 |  | 0.95  (0.91 - 1.00) | 3.07E-02 | 4.43E-01 |  | 0.97  (0.91 - 1.03) | 2.97E-01 | 8.24E-01 |  | 1.01  (0.96 - 1.06) | 6.51E-01 | 6.47E-01 |
|  | Simple mode | 7 |  | 0.91  (0.83 - 1.00) | 9.18E-02 |  |  | 0.94  (0.83 - 1.05) | 3.16E-01 |  |  | 1.04  (0.95 - 1.13) | 4.36E-01 |  |
|  | Weighted mode | 7 |  | 0.97  (0.90 - 1.05) | 5.32E-01 |  |  | 0.94  (0.84 - 1.05) | 2.85E-01 |  |  | 1.03  (0.95 - 1.11) | 5.25E-01 |  |
|  | RAPS | 7 |  | 0.95  (0.90 - 1.00) | 5.97E-02 |  |  | 0.97  (0.9 - 1.03) | 3.12E-01 |  |  | 1.01  (0.96 - 1.06) | 6.61E-01 |  |
| *Continued on next page* | | | | | | | | | | | | | | |
|  |  |  |  |  |  |  |  |  |  |  |  |  |  |  |
|  |  |  |  |  |  |  |  |  |  |  |  |  |  |  |
| **Table S11. *Continued*** | | | | | | | | | | | | | | |
| Fe | MR Egger | 86 |  | 1.02  (0.94 - 1.10) | 7.01E-01 | 3.96E-01 |  | 1.14  (1.00 - 1.3) | 5.26E-02 | 7.87E-03 |  | 1.05  (0.96 - 1.14) | 2.88E-01 | 1.61E-01 |
|  | Weighted median | 86 |  | 1.02  (0.95 - 1.09) | 6.75E-01 |  |  | 0.95  (0.83 - 1.08) | 4.08E-01 |  |  | 1.07  (0.99 - 1.16) | 1.05E-01 |  |
|  | IVW | 86 |  | 1.00  (0.95 - 1.05) | 9.59E-01 | 4.16E-01 |  | 1.02  (0.93 - 1.12) | 6.01E-01 | 2.89E-03 |  | 1.04  (0.98 - 1.11) | 1.58E-01 | 1.80E-01 |
|  | Simple mode | 86 |  | 1.00  (0.84 - 1.19) | 9.86E-01 |  |  | 0.9  (0.62 - 1.3) | 5.85E-01 |  |  | 1.07  (0.87 - 1.32) | 5.20E-01 |  |
|  | Weighted mode | 86 |  | 1.01  (0.95 - 1.08) | 6.66E-01 |  |  | 1.07  (0.96 - 1.18) | 2.29E-01 |  |  | 1.05  (0.98 - 1.13) | 1.51E-01 |  |
|  | RAPS | 86 |  | 1.00  (0.95 - 1.06) | 9.78E-01 |  |  | 1.04  (0.94 - 1.15) | 4.76E-01 |  |  | 1.05  (0.99 - 1.11) | 1.22E-01 |  |
| Se QIMR | MR Egger | 5 |  | 1.02  (0.95 - 1.1) | 6.22E-01 | 7.35E-01 |  | 1.06  (0.94 - 1.19) | 4.15E-01 | 3.43E-01 |  | 1.03  (0.90 - 1.19) | 6.88E-01 | 2.79E-02 |
|  | Weighted median | 5 |  | 0.99  (0.95 - 1.04) | 7.17E-01 |  |  | 1.04  (0.98 - 1.12) | 2.13E-01 |  |  | 1.02  (0.98 - 1.08) | 3.32E-01 |  |
|  | IVW | 5 |  | 0.99  (0.96 - 1.03) | 6.99E-01 | 7.36E-01 |  | 1.04  (0.98 - 1.10) | 1.59E-01 | 4.86E-01 |  | 1.01  (0.95 - 1.08) | 6.46E-01 | 5.31E-02 |
|  | Simple mode | 5 |  | 0.97  (0.90 - 1.04) | 4.24E-01 |  |  | 1.07  (0.98 - 1.17) | 2.26E-01 |  |  | 1.02  (0.95 - 1.08) | 6.80E-01 |  |
|  | Weighted mode | 5 |  | 1.02  (0.96 - 1.08) | 6.23E-01 |  |  | 1.05  (0.96 - 1.14) | 3.32E-01 |  |  | 1.02  (0.97 - 1.07) | 4.44E-01 |  |
|  | RAPS | 5 |  | 0.99  (0.95 - 1.03) | 7.07E-01 |  |  | 1.04  (0.98 - 1.10) | 1.79E-01 |  |  | 1.02  (0.96 - 1.07) | 5.25E-01 |  |
| *Continued on next page* | | | | | | | | | | | | | | |
|  |  |  |  |  |  |  |  |  |  |  |  |  |  |  |
| **Table S11. *Continued*** | | | | | | | | | | | | | | |
| Se ALSPAC | MR Egger | 6 |  | 0.86  (0.74 - 1.01) | 1.42E-01 | 2.70E-01 |  | 1.03  (0.85 - 1.26) | 7.65E-01 | 7.50E-01 |  | 1.03  (0.89 - 1.19) | 7.30E-01 | 5.75E-01 |
|  | Weighted median | 6 |  | 0.93  (0.87 - 0.98) | 1.02E-02 |  |  | 1.01  (0.93 - 1.09) | 7.79E-01 |  |  | 1.03  (0.97 - 1.10) | 2.81E-01 |  |
|  | IVW | 6 |  | 0.96  (0.91 - 1.02) | 2.12E-01 | 1.61E-01 |  | 1.02  (0.96 - 1.09) | 4.59E-01 | 8.59E-01 |  | 1.04  (0.99 - 1.09) | 1.03E-01 | 7.12E-01 |
|  | Simple mode | 6 |  | 0.93  (0.85 - 1.02) | 2.02E-01 |  |  | 1.02  (0.92 - 1.14) | 7.32E-01 |  |  | 1.02  (0.94 - 1.12) | 6.09E-01 |  |
|  | Weighted mode | 6 |  | 0.93  (0.87 - 0.99) | 5.97E-02 |  |  | 1.01  (0.92 - 1.12) | 7.89E-01 |  |  | 1.03  (0.96 - 1.10) | 4.32E-01 |  |
|  | RAPS | 6 |  | 0.96  (0.9 - 1.02) | 1.50E-01 |  |  | 1.02  (0.96 - 1.09) | 4.72E-01 |  |  | 1.04  (0.99 - 1.09) | 1.17E-01 |  |
| Zn | MR Egger | 7 |  | 1.08  (0.91 - 1.29) | 4.26E-01 | 2.04E-01 |  | 1.14  (0.92 - 1.40) | 2.91E-01 | 4.12E-01 |  | 1.06  (0.90 - 1.23) | 5.25E-01 | 4.34E-01 |
|  | Weighted median | 7 |  | 1.01  (0.95 - 1.06) | 8.09E-01 |  |  | 0.95  (0.88 - 1.03) | 2.04E-01 |  |  | 1.03  (0.98 - 1.09) | 2.44E-01 |  |
|  | IVW | 7 |  | 1.01  (0.96 - 1.06) | 6.95E-01 | 2.28E-01 |  | 0.96  (0.90 - 1.02) | 1.85E-01 | 2.59E-01 |  | 1.01  (0.97 - 1.06) | 4.79E-01 | 5.28E-01 |
|  | Simple mode | 7 |  | 1.07  (0.97 - 1.17) | 2.27E-01 |  |  | 0.97  (0.87 - 1.08) | 5.54E-01 |  |  | 1.04  (0.96 - 1.13) | 4.11E-01 |  |
|  | Weighted mode | 7 |  | 1.00  (0.93 - 1.07) | 9.76E-01 |  |  | 0.95  (0.87 - 1.04) | 3.02E-01 |  |  | 1.04  (0.97 - 1.11) | 3.51E-01 |  |
|  | RAPS | 7 |  | 1.02  (0.97 - 1.07) | 5.24E-01 |  |  | 0.96  (0.90 - 1.02) | 1.56E-01 |  |  | 1.02  (0.97 - 1.06) | 4.68E-01 |  |
| *Continued on next page* | | | | | | | | | | | | | | |
|  |  |  |  |  |  |  |  |  |  |  |  |  |  |  |
| **Table S11. *Continued*** | | | | | | | | | | | | | | |
| Vitamin B12 | MR Egger | 10 |  | 0.89  (0.71 - 1.10) | 3.11E-01 | 5.89E-04 |  | 1.08  (0.89 - 1.3) | 4.83E-01 | 2.17E-01 |  | 1.01  (0.87 - 1.17) | 9.29E-01 | 1.57E-01 |
|  | Weighted median | 10 |  | 0.96  (0.87 - 1.07) | 4.77E-01 |  |  | 1.10  (0.98 - 1.22) | 1.08E-01 |  |  | 1.03  (0.94 - 1.12) | 5.64E-01 |  |
|  | IVW | 10 |  | 0.97  (0.87 - 1.07) | 5.49E-01 | 3.92E-04 |  | 1.09  (1.00 - 1.19) | 5.98E-02 | 2.92E-01 |  | 0.99  (0.93 - 1.06) | 8.74E-01 | 2.18E-01 |
|  | Simple mode | 10 |  | 0.93  (0.75 - 1.14) | 4.83E-01 |  |  | 1.11  (0.94 - 1.32) | 2.57E-01 |  |  | 1.07  (0.93 - 1.23) | 3.84E-01 |  |
|  | Weighted mode | 10 |  | 0.86  (0.66 - 1.1) | 2.62E-01 |  |  | 1.11  (0.98 - 1.25) | 1.29E-01 |  |  | 1.07  (0.94 - 1.21) | 3.51E-01 |  |
|  | RAPS | 10 |  | 0.98  (0.89 - 1.08) | 6.79E-01 |  |  | 1.08  (0.99 - 1.19) | 9.60E-02 |  |  | 1.00  (0.93 - 1.08) | 8.99E-01 |  |
| Vitamin C | MR Egger | 19 |  | 0.89  (0.74 - 1.06) | 1.94E-01 | 1.26E-01 |  | 1.07  (0.80 - 1.42) | 6.71E-01 | 7.59E-03 |  | 1.03  (0.88 - 1.20) | 7.21E-01 | 3.87E-01 |
|  | Weighted median | 19 |  | 0.89  (0.79 - 1.00) | 5.97E-02 |  |  | 0.98  (0.82 - 1.18) | 8.55E-01 |  |  | 1.05  (0.93 - 1.18) | 4.64E-01 |  |
|  | IVW | 19 |  | 0.98  (0.88 - 1.08) | 6.28E-01 | 9.68E-02 |  | 0.95  (0.80 - 1.12) | 5.44E-01 | 6.65E-03 |  | 0.97  (0.89 - 1.06) | 5.01E-01 | 3.96E-01 |
|  | Simple mode | 19 |  | 0.94  (0.76 - 1.16) | 5.43E-01 |  |  | 0.98  (0.71 - 1.35) | 9.00E-01 |  |  | 0.98  (0.78 - 1.24) | 8.84E-01 |  |
|  | Weighted mode | 19 |  | 0.90  (0.80 - 1.01) | 8.67E-02 |  |  | 1.01  (0.85 - 1.20) | 9.20E-01 |  |  | 1.02  (0.90 - 1.16) | 7.35E-01 |  |
|  | RAPS | 19 |  | 0.95  (0.87 - 1.05) | 3.28E-01 |  |  | 0.96  (0.82 - 1.12) | 5.97E-01 |  |  | 0.98  (0.89 - 1.07) | 5.96E-01 |  |
| *Continued on next page* | | | | | | | | | | | | | | |
|  |  |  |  |  |  |  |  |  |  |  |  |  |  |  |
|  |  |  |  |  |  |  |  |  |  |  |  |  |  |  |
| **Table S11. *Continued*** | | | | | | | | | | | | | | |
| Vitamin D | MR Egger | 69 |  | 1.03  (0.90 - 1.17) | 7.02E-01 | 6.06E-01 |  | 1.08  (0.88 - 1.33) | 4.85E-01 | 8.11E-02 |  | 0.98  (0.84 - 1.15) | 8.42E-01 | 1.91E-02 |
|  | Weighted median | 69 |  | 1.08  (0.95 - 1.24) | 2.28E-01 |  |  | 1.12  (0.93 - 1.35) | 2.25E-01 |  |  | 0.98  (0.86 - 1.12) | 7.87E-01 |  |
|  | IVW | 69 |  | 1.11  (1.02 - 1.21) | 2.00E-02 | 5.56E-01 |  | 1.05  (0.92 - 1.20) | 4.86E-01 | 9.27E-02 |  | 1.00  (0.90 - 1.12) | 9.43E-01 | 2.27E-02 |
|  | Simple mode | 69 |  | 1.07  (0.85 - 1.37) | 5.59E-01 |  |  | 1.08  (0.74 - 1.57) | 7.00E-01 |  |  | 0.91  (0.66 - 1.27) | 5.88E-01 |  |
|  | Weighted mode | 69 |  | 1.05  (0.93 - 1.19) | 4.37E-01 |  |  | 1.09  (0.93 - 1.28) | 2.83E-01 |  |  | 0.96  (0.85 - 1.08) | 4.96E-01 |  |
|  | RAPS | 69 |  | 1.10  (1.00 - 1.20) | 4.00E-02 |  |  | 1.06  (0.92 - 1.22) | 3.92E-01 |  |  | 0.99  (0.89 - 1.10) | 8.04E-01 |  |
| ^a^ SNPs with *r^2^* < 0.01 within 10,000 kb windows and *P* ≤ 5E-06 were used as genetic instruments for the exposure.  ^b^ The odds ratios (OR) correspond to a 1 standard deviation increase in the concentration of the micronutrient, expect for Cu Jager et al.  ^c^ The beta-coefficient from Jäger et al. was converted from computed Z-scores.  Abbreviations: Cu: Copper, Fe: Iron, IVW: inverse-variance weighted, RAPS: robust adjusted profile score, Se: Selenium, Zn: Zinc | | | | | | | | | | | | | | |

| **Table S12.** Secondary mendelian randomization analyses of copper as risk factors on the risk of gastrointestinal infections, where rs12582659 was removed. | | | | | | | | | | | | | | |
| --- | --- | --- | --- | --- | --- | --- | --- | --- | --- | --- | --- | --- | --- | --- |
|  |  |  |  | **Gastrointestinal infections** | | |  | **Pneumonia** | | |  | **Urinary tract infections** | | |
| Exposure^a^ | Method | **Number of SNPs** |  | **OR^b^**  **(95% CI)** | ***P* value** | **Cochran's Q** |  | **OR^b^**  **(95% CI)** | ***P* value** | **Cochran's Q** |  | **OR^b^**  **(95% CI)** | ***P* value** | **Cochran's Q** |
| Cu Evans et al. | MR Egger | 5 |  | 0.78  (0.68 - 0.90) | 3.91E-01 | 6.09E-01 |  | 0.89  (0.73 - 1.08) | 3.20E-01 | 8.17E-01 |  | 0.94  (0.81 - 1.09) | 8.37E-01 | 4.27E-01 |
|  | Weighted median | 5 |  | 0.87  (0.87 - 1.00) | 3.72E-02 |  |  | 0.69  (0.89 - 1.03) | 2.62E-01 |  |  | 1.00  (0.94 - 1.06) | 9.58E-01 |  |
|  | IVW | 5 |  | 0.97  (0.90 - 1.05) | 4.35E-01 | 1.47E-02 |  | 0.97  (0.91 - 1.03) | 3.55E-01 | 7.651E-01 |  | 1.02  (0.98 - 1.07) | 3.659E-01 | 3.75E-01 |
|  | Simple mode | 5 |  | 1.06  (0.91 - 1.24) | 2.63E-01 |  |  | 0.96  (0.87 - 1.05) | 4.41E-01 |  |  | 1.00  (0.98 - 1.08) | 9.44E-01 |  |
|  | Weighted mode | 5 |  | 0.92  (0.86 - 0.98) | 5.96E-02 |  |  | 0.96  (0.88 - 1.04) | 9.09E-01 |  |  | 1.00  (0.93 - 1.06) | 9.14E-01 |  |
|  | RAPS | 5 |  | 0.96  (0.89 - 1.04) | 3.27E-01 |  |  | 0.97  (0.91 - 1.04) | 7.09E-01 |  |  | 1.02  (0.97 - 1.07) | 4.28E-01 |  |
| ^a^ SNPs with *r^2^* < 0.01 within 10,000 kb windows and *P* ≤ 5E-06 were used as genetic instruments for the exposure.  ^b^ The odds ratios (OR) correspond to a 1 standard deviation increase in the concentration of the micronutrient.  Abbreviations: Cu: Copper, IVW: inverse-variance weighted, RAPS: robust adjusted profile score. | | | | | | | | | | | | | | |

| **Table S21.** IVW MR regression results for the leave one SNP out analysis in the Mendelian randomization analyses of micronutrients. | | | | | | | | | | |
| --- | --- | --- | --- | --- | --- | --- | --- | --- | --- | --- |
|  |  |  | **Gastrointestinal infections** | |  | **Pneumonia** | |  | **Urinary tract infections** | |
| **Exposure^a^** | **SNP left out** |  | **OR^b^ (95% CI)** | ***P* value** |  | **OR^b^ (95% CI)** | ***P* value** |  | **OR^b^ (95% CI)** | ***P* value** |
| Fe | rs12718598 |  | 1.00 (0.93 - 1.08) | 9.49E-01 |  | 1.02 (0.87 - 1.19) | 8.37E-01 |  | 1.02 (0.87 - 1.19) | 8.37E-01 |
|  | rs13007705 |  | 1.00 (0.93 - 1.08) | 9.70E-01 |  | 1.02 (0.87 - 1.19) | 8.53E-01 |  | 1.02 (0.87 - 1.19) | 8.53E-01 |
|  | rs1800562 |  | 1.00 (0.92 - 1.09) | 9.43E-01 |  | 0.92 (0.79 - 1.07) | 2.75E-01 |  | 0.92 (0.79 - 1.07) | 2.75E-01 |
|  | rs2005682 |  | 1.00 (0.93 - 1.07) | 9.62E-01 |  | 1.01 (0.86 - 1.18) | 9.09E-01 |  | 1.01 (0.86 - 1.18) | 9.09E-01 |
|  | rs2228145 |  | 1.00 (0.93 - 1.08) | 9.06E-01 |  | 1.00 (0.88 - 1.13) | 9.54E-01 |  | 1.00 (0.88 - 1.13) | 9.54E-01 |
|  | rs4854760 |  | 1.00 (0.93 - 1.08) | 9.82E-01 |  | 1.02 (0.87 - 1.20) | 8.18E-01 |  | 1.02 (0.87 - 1.20) | 8.18E-01 |
|  | rs57659670 |  | 1.00 (0.93 - 1.08) | 9.60E-01 |  | 1.01 (0.86 - 1.19) | 8.70E-01 |  | 1.01 (0.86 - 1.19) | 8.70E-01 |
|  | rs7385804 |  | 1.00 (0.93 - 1.08) | 9.81E-01 |  | 1.05 (0.91 - 1.20) | 5.08E-01 |  | 1.05 (0.91 - 1.20) | 5.08E-01 |
|  | rs7630745 |  | 1.00 (0.93 - 1.07) | 9.04E-01 |  | 1.01 (0.86 - 1.18) | 9.08E-01 |  | 1.01 (0.86 - 1.18) | 9.08E-01 |
|  | rs77262773 |  | 1.00 (0.93 - 1.08) | 9.22E-01 |  | 1.01 (0.87 - 1.19) | 8.54E-01 |  | 1.01 (0.87 - 1.19) | 8.54E-01 |
|  | rs855791 |  | 0.98 (0.89 - 1.09) | 7.68E-01 |  | 1.09 (0.88 - 1.35) | 4.39E-01 |  | 1.09 (0.88 - 1.35) | 4.39E-01 |
|  | rs9399136 |  | 1.00 (0.94 - 1.08) | 8.94E-01 |  | 1.02 (0.87 - 1.19) | 8.28E-01 |  | 1.02 (0.87 - 1.19) | 8.28E-01 |
| Vitamin B12 | rs1131603 |  | 0.96 (0.85 - 1.08) | 4.86E-01 |  | 1.07 (0.97 - 1.17) | 1.72E-01 |  | 0.99 (0.92 - 1.07) | 8.50E-01 |
|  | rs1141321 |  | 0.98 (0.87 - 1.10) | 6.81E-01 |  | 1.10 (1.01 - 1.20) | 2.64E-02 |  | 1.01 (0.94 - 1.08) | 8.44E-01 |
|  | rs1801222 |  | 0.95 (0.84 - 1.06) | 3.25E-01 |  | 1.09 (0.98 - 1.21) | 1.22E-01 |  | 0.98 (0.91 - 1.05) | 5.83E-01 |
|  | rs2270655 |  | 0.97 (0.86 - 1.09) | 6.27E-01 |  | 1.09 (0.98 - 1.20) | 1.05E-01 |  | 0.99 (0.92 - 1.07) | 8.60E-01 |
|  | rs2336573 |  | 0.98 (0.87 - 1.11) | 7.89E-01 |  | 1.09 (0.98 - 1.21) | 1.32E-01 |  | 1.00 (0.92 - 1.09) | 9.58E-01 |
|  | rs34324219 |  | 0.94 (0.83 - 1.06) | 3.32E-01 |  | 1.12 (1.01 - 1.24) | 3.65E-02 |  | 0.98 (0.90 - 1.06) | 5.51E-01 |
|  | rs3742801 |  | 0.96 (0.86 - 1.07) | 4.59E-01 |  | 1.08 (0.98 - 1.19) | 1.09E-01 |  | 1.00 (0.92 - 1.08) | 9.19E-01 |
|  | rs41281112 |  | 0.97 (0.86 - 1.09) | 6.27E-01 |  | 1.10 (1.01 - 1.21) | 3.19E-02 |  | 0.99 (0.92 - 1.07) | 8.21E-01 |
|  | rs602662 |  | 1.05 (0.96 - 1.15) | 2.63E-01 |  | 1.06 (0.94 - 1.18) | 3.43E-01 |  | 1.04 (0.97 - 1.12) | 2.42E-01 |
| *Continued on next page* | | | | | | | | | | |
|  |  |  |  |  |  |  |  |  |  |  |
|  |  |  |  |  |  |  |  |  |  |  |
|  |  |  |  |  |  |  |  |  |  |  |
| **Table S21. *Continued*** | | | | | | | | | | |
| Vitamin C | rs10051765 |  | 0.96 (0.85 - 1.08) | 4.65E-01 |  | 0.98 (0.89 - 1.09) | 7.44E-01 |  | 0.98 (0.89 - 1.09) | 7.44E-01 |
|  | rs10136000 |  | 0.97 (0.86 - 1.08) | 5.60E-01 |  | 0.98 (0.89 - 1.09) | 7.65E-01 |  | 0.98 (0.89 - 1.09) | 7.65E-01 |
|  | rs117885456 |  | 0.94 (0.84 - 1.06) | 3.21E-01 |  | 0.98 (0.88 - 1.10) | 7.59E-01 |  | 0.98 (0.88 - 1.10) | 7.59E-01 |
|  | rs13028225 |  | 0.95 (0.85 - 1.07) | 4.15E-01 |  | 0.97 (0.86 - 1.09) | 5.83E-01 |  | 0.97 (0.86 - 1.09) | 5.83E-01 |
|  | rs174547 |  | 0.95 (0.85 - 1.06) | 3.54E-01 |  | 0.96 (0.85 - 1.08) | 5.09E-01 |  | 0.96 (0.85 - 1.08) | 5.09E-01 |
|  | rs2559850 |  | 0.97 (0.87 - 1.10) | 6.66E-01 |  | 0.97 (0.86 - 1.10) | 6.30E-01 |  | 0.97 (0.86 - 1.10) | 6.30E-01 |
|  | rs33972313 |  | 1.02 (0.88 - 1.17) | 7.93E-01 |  | 0.90 (0.78 - 1.04) | 1.45E-01 |  | 0.90 (0.78 - 1.04) | 1.45E-01 |
|  | rs56738967 |  | 0.93 (0.84 - 1.03) | 1.44E-01 |  | 0.97 (0.86 - 1.09) | 5.69E-01 |  | 0.97 (0.86 - 1.09) | 5.69E-01 |
|  | rs6693447 |  | 0.95 (0.84 - 1.07) | 3.81E-01 |  | 0.97 (0.86 - 1.09) | 5.78E-01 |  | 0.97 (0.86 - 1.09) | 5.78E-01 |
|  | rs9895661 |  | 0.96 (0.85 - 1.08) | 4.85E-01 |  | 0.95 (0.85 - 1.06) | 3.25E-01 |  | 0.95 (0.85 - 1.06) | 3.25E-01 |
| Vitamin D | rs1011468 |  | 1.11 (1.02 - 1.21) | 1.53E-02 |  | 1.07 (0.94 - 1.21) | 3.29E-01 |  | 1.01 (0.91 - 1.12) | 8.47E-01 |
|  | rs1047891 |  | 1.11 (1.02 - 1.21) | 1.80E-02 |  | 1.06 (0.92 - 1.21) | 4.39E-01 |  | 1.01 (0.91 - 1.12) | 8.60E-01 |
|  | rs10500209 |  | 1.11 (1.02 - 1.21) | 1.89E-02 |  | 1.04 (0.91 - 1.20) | 5.40E-01 |  | 1.00 (0.90 - 1.12) | 9.60E-01 |
|  | rs10818769 |  | 1.11 (1.02 - 1.21) | 2.08E-02 |  | 1.05 (0.92 - 1.21) | 4.70E-01 |  | 1.01 (0.90 - 1.12) | 9.09E-01 |
|  | rs10832218 |  | 1.11 (1.02 - 1.21) | 2.04E-02 |  | 1.05 (0.91 - 1.20) | 5.32E-01 |  | 0.99 (0.89 - 1.10) | 8.00E-01 |
|  | rs10832289 |  | 1.11 (1.02 - 1.21) | 1.74E-02 |  | 1.05 (0.91 - 1.21) | 5.16E-01 |  | 1.00 (0.90 - 1.12) | 9.44E-01 |
|  | rs10859995 |  | 1.12 (1.03 - 1.23) | 9.76E-03 |  | 1.06 (0.92 - 1.22) | 3.97E-01 |  | 1.01 (0.91 - 1.13) | 8.24E-01 |
|  | rs10887718 |  | 1.11 (1.01 - 1.20) | 2.27E-02 |  | 1.05 (0.91 - 1.20) | 4.96E-01 |  | 1.01 (0.90 - 1.12) | 9.20E-01 |
|  | rs11127048 |  | 1.11 (1.01 - 1.21) | 2.17E-02 |  | 1.05 (0.92 - 1.21) | 4.49E-01 |  | 1.01 (0.91 - 1.12) | 8.76E-01 |
|  | rs111529171 |  | 1.11 (1.02 - 1.21) | 1.56E-02 |  | 1.06 (0.93 - 1.21) | 3.59E-01 |  | 1.00 (0.90 - 1.11) | 9.91E-01 |
|  | rs11264360 |  | 1.11 (1.02 - 1.21) | 2.05E-02 |  | 1.04 (0.91 - 1.20) | 5.32E-01 |  | 1.00 (0.90 - 1.11) | 9.72E-01 |
|  | rs1149605 |  | 1.10 (1.01 - 1.20) | 3.04E-02 |  | 1.05 (0.91 - 1.20) | 5.26E-01 |  | 1.01 (0.91 - 1.12) | 8.88E-01 |
|  | rs12123821 |  | 1.11 (1.01 - 1.21) | 2.33E-02 |  | 1.05 (0.91 - 1.20) | 5.19E-01 |  | 1.01 (0.91 - 1.12) | 8.72E-01 |
|  | rs1229984 |  | 1.11 (1.02 - 1.21) | 1.60E-02 |  | 1.05 (0.92 - 1.21) | 4.70E-01 |  | 1.00 (0.90 - 1.11) | 9.69E-01 |
|  | rs12317268 |  | 1.11 (1.02 - 1.21) | 1.94E-02 |  | 1.04 (0.91 - 1.20) | 5.44E-01 |  | 1.01 (0.91 - 1.12) | 8.94E-01 |
|  | rs12803256 |  | 1.11 (1.00 - 1.23) | 4.10E-02 |  | 1.01 (0.86 - 1.18) | 9.26E-01 |  | 1.02 (0.90 - 1.15) | 7.78E-01 |
|  | rs12997242 |  | 1.11 (1.02 - 1.21) | 1.97E-02 |  | 1.05 (0.91 - 1.20) | 5.06E-01 |  | 1.00 (0.90 - 1.12) | 9.41E-01 |
| *Continued on next page* | | | | | | | | | | |
|  |  |  |  |  |  |  |  |  |  |  |
| **Table S21. *Continued*** | | | | | | | | | | |
| Vitamin D | rs157595 |  | 1.11 (1.02 - 1.21) | 1.79E-02 |  | 1.04 (0.91 - 1.20) | 5.30E-01 |  | 1.00 (0.90 - 1.11) | 9.79E-01 |
|  | rs17765311 |  | 1.11 (1.01 - 1.20) | 2.30E-02 |  | 1.05 (0.92 - 1.21) | 4.79E-01 |  | 1.01 (0.91 - 1.12) | 8.54E-01 |
|  | rs1800588 |  | 1.11 (1.01 - 1.21) | 2.18E-02 |  | 1.06 (0.93 - 1.22) | 3.95E-01 |  | 1.00 (0.90 - 1.11) | 9.73E-01 |
|  | rs1800775 |  | 1.11 (1.02 - 1.21) | 1.76E-02 |  | 1.05 (0.91 - 1.20) | 5.31E-01 |  | 1.00 (0.90 - 1.12) | 9.56E-01 |
|  | rs1858889 |  | 1.11 (1.02 - 1.21) | 1.58E-02 |  | 1.05 (0.91 - 1.20) | 4.98E-01 |  | 1.00 (0.90 - 1.12) | 9.56E-01 |
|  | rs1972994 |  | 1.10 (1.01 - 1.20) | 2.52E-02 |  | 1.05 (0.91 - 1.20) | 5.18E-01 |  | 1.00 (0.90 - 1.12) | 9.36E-01 |
|  | rs2011425 |  | 1.11 (1.02 - 1.21) | 2.11E-02 |  | 1.06 (0.93 - 1.22) | 3.86E-01 |  | 1.01 (0.91 - 1.13) | 7.97E-01 |
|  | rs2037511 |  | 1.11 (1.02 - 1.21) | 1.96E-02 |  | 1.05 (0.92 - 1.21) | 4.52E-01 |  | 1.00 (0.90 - 1.12) | 9.28E-01 |
|  | rs2074735 |  | 1.10 (1.01 - 1.20) | 2.43E-02 |  | 1.06 (0.92 - 1.21) | 4.04E-01 |  | 1.01 (0.91 - 1.12) | 8.62E-01 |
|  | rs2229742 |  | 1.10 (1.01 - 1.20) | 2.38E-02 |  | 1.05 (0.91 - 1.20) | 4.97E-01 |  | 1.00 (0.90 - 1.11) | 9.88E-01 |
|  | rs261291 |  | 1.10 (1.01 - 1.20) | 3.48E-02 |  | 1.04 (0.91 - 1.20) | 5.37E-01 |  | 0.99 (0.89 - 1.10) | 9.06E-01 |
|  | rs2762942 |  | 1.11 (1.02 - 1.21) | 2.04E-02 |  | 1.05 (0.91 - 1.21) | 4.96E-01 |  | 1.01 (0.90 - 1.12) | 9.21E-01 |
|  | rs2847500 |  | 1.11 (1.02 - 1.21) | 1.72E-02 |  | 1.06 (0.92 - 1.21) | 4.25E-01 |  | 1.00 (0.90 - 1.11) | 9.66E-01 |
|  | rs2909218 |  | 1.10 (1.01 - 1.20) | 2.43E-02 |  | 1.05 (0.92 - 1.21) | 4.48E-01 |  | 1.00 (0.90 - 1.11) | 9.97E-01 |
|  | rs2934744 |  | 1.11 (1.02 - 1.21) | 2.12E-02 |  | 1.04 (0.91 - 1.20) | 5.50E-01 |  | 1.00 (0.90 - 1.11) | 9.80E-01 |
|  | rs34726834 |  | 1.11 (1.02 - 1.21) | 1.51E-02 |  | 1.05 (0.92 - 1.21) | 4.83E-01 |  | 1.00 (0.90 - 1.12) | 9.31E-01 |
|  | rs3750296 |  | 1.11 (1.02 - 1.21) | 1.91E-02 |  | 1.05 (0.91 - 1.20) | 4.99E-01 |  | 1.00 (0.90 - 1.11) | 9.89E-01 |
|  | rs3768013 |  | 1.11 (1.02 - 1.21) | 2.11E-02 |  | 1.04 (0.91 - 1.19) | 5.55E-01 |  | 1.00 (0.90 - 1.11) | 9.84E-01 |
|  | rs3814995 |  | 1.11 (1.01 - 1.21) | 2.23E-02 |  | 1.05 (0.92 - 1.21) | 4.69E-01 |  | 0.99 (0.90 - 1.10) | 9.23E-01 |
|  | rs3822868 |  | 1.11 (1.02 - 1.21) | 1.64E-02 |  | 1.04 (0.91 - 1.19) | 5.57E-01 |  | 1.01 (0.90 - 1.12) | 9.22E-01 |
|  | rs523583 |  | 1.11 (1.02 - 1.21) | 1.92E-02 |  | 1.04 (0.91 - 1.20) | 5.33E-01 |  | 1.00 (0.90 - 1.12) | 9.40E-01 |
|  | rs532436 |  | 1.11 (1.02 - 1.21) | 2.03E-02 |  | 1.05 (0.92 - 1.21) | 4.44E-01 |  | 1.00 (0.90 - 1.12) | 9.33E-01 |
|  | rs56044892 |  | 1.10 (1.01 - 1.20) | 2.35E-02 |  | 1.05 (0.91 - 1.20) | 4.91E-01 |  | 1.00 (0.90 - 1.11) | 9.93E-01 |
|  | rs57631352 |  | 1.11 (1.02 - 1.21) | 2.09E-02 |  | 1.05 (0.92 - 1.21) | 4.44E-01 |  | 1.00 (0.90 - 1.11) | 9.74E-01 |
|  | rs577185477 |  | 1.14 (1.04 - 1.26) | 6.68E-03 |  | 1.07 (0.92 - 1.25) | 3.61E-01 |  | 1.01 (0.90 - 1.13) | 9.09E-01 |
|  | rs58073039 |  | 1.10 (1.01 - 1.20) | 2.79E-02 |  | 1.04 (0.91 - 1.19) | 5.48E-01 |  | 1.00 (0.90 - 1.12) | 9.52E-01 |
|  | rs58542926 |  | 1.11 (1.01 - 1.21) | 2.29E-02 |  | 1.05 (0.91 - 1.21) | 4.86E-01 |  | 1.01 (0.91 - 1.12) | 7.92E-01 |
| *Continued on next page* | | | | | | | | | | |
|  |  |  |  |  |  |  |  |  |  |  |
| **Table S21. *Continued*** | | | | | | | | | | |
| Vitamin D | rs6123359 |  | 1.11 (1.01 - 1.21) | 2.33E-02 |  | 1.04 (0.91 - 1.20) | 5.51E-01 |  | 1.00 (0.90 - 1.11) | 9.33E-01 |
|  | rs6127099 |  | 1.10 (1.01 - 1.20) | 3.04E-02 |  | 1.04 (0.91 - 1.20) | 5.75E-01 |  | 0.99 (0.89 - 1.10) | 8.57E-01 |
|  | rs62007299 |  | 1.10 (1.01 - 1.20) | 3.06E-02 |  | 1.05 (0.92 - 1.21) | 4.64E-01 |  | 1.01 (0.91 - 1.12) | 8.64E-01 |
|  | rs6438900 |  | 1.11 (1.02 - 1.21) | 1.94E-02 |  | 1.05 (0.92 - 1.21) | 4.84E-01 |  | 1.00 (0.90 - 1.11) | 9.61E-01 |
|  | rs6698680 |  | 1.11 (1.02 - 1.21) | 2.12E-02 |  | 1.05 (0.92 - 1.21) | 4.79E-01 |  | 1.00 (0.90 - 1.12) | 9.32E-01 |
|  | rs6724965 |  | 1.10 (1.01 - 1.20) | 3.11E-02 |  | 1.05 (0.91 - 1.20) | 5.23E-01 |  | 1.00 (0.90 - 1.11) | 9.71E-01 |
|  | rs6773343 |  | 1.11 (1.02 - 1.21) | 1.61E-02 |  | 1.04 (0.91 - 1.20) | 5.28E-01 |  | 1.00 (0.90 - 1.12) | 9.27E-01 |
|  | rs73015021 |  | 1.11 (1.02 - 1.21) | 1.85E-02 |  | 1.05 (0.91 - 1.20) | 5.10E-01 |  | 1.00 (0.90 - 1.12) | 9.54E-01 |
|  | rs7519574 |  | 1.10 (1.01 - 1.20) | 2.39E-02 |  | 1.05 (0.91 - 1.20) | 5.26E-01 |  | 1.00 (0.90 - 1.12) | 9.63E-01 |
|  | rs7528419 |  | 1.11 (1.02 - 1.21) | 1.86E-02 |  | 1.06 (0.92 - 1.21) | 4.35E-01 |  | 1.01 (0.90 - 1.12) | 9.27E-01 |
|  | rs7569755 |  | 1.11 (1.02 - 1.21) | 1.66E-02 |  | 1.05 (0.92 - 1.21) | 4.57E-01 |  | 1.01 (0.90 - 1.12) | 9.05E-01 |
|  | rs7699711 |  | 1.10 (1.01 - 1.20) | 2.91E-02 |  | 1.05 (0.91 - 1.21) | 4.95E-01 |  | 1.01 (0.91 - 1.12) | 8.83E-01 |
|  | rs7718395 |  | 1.10 (1.01 - 1.20) | 2.69E-02 |  | 1.04 (0.91 - 1.20) | 5.34E-01 |  | 1.01 (0.90 - 1.12) | 9.12E-01 |
|  | rs77924615 |  | 1.10 (1.01 - 1.20) | 2.32E-02 |  | 1.05 (0.91 - 1.20) | 5.26E-01 |  | 1.01 (0.91 - 1.12) | 9.01E-01 |
|  | rs7828742 |  | 1.11 (1.02 - 1.21) | 1.64E-02 |  | 1.04 (0.91 - 1.20) | 5.44E-01 |  | 1.00 (0.90 - 1.12) | 9.40E-01 |
|  | rs78649910 |  | 1.10 (1.01 - 1.20) | 2.44E-02 |  | 1.05 (0.91 - 1.20) | 5.09E-01 |  | 1.01 (0.91 - 1.12) | 8.75E-01 |
|  | rs8018720 |  | 1.10 (1.01 - 1.20) | 3.04E-02 |  | 1.05 (0.91 - 1.21) | 4.91E-01 |  | 0.99 (0.89 - 1.10) | 8.86E-01 |
|  | rs804280 |  | 1.11 (1.01 - 1.21) | 2.27E-02 |  | 1.05 (0.91 - 1.21) | 4.88E-01 |  | 1-00 (0.90 - 1.12) | 9.45E-01 |
|  | rs8063706 |  | 1.11 (1.02 - 1.21) | 1.99E-02 |  | 1.05 (0.92 - 1.21) | 4.51E-01 |  | 1.00 (0.90 - 1.12) | 9.50E-01 |
|  | rs8091117 |  | 1.11 (1.02 - 1.21) | 1.97E-02 |  | 1.05 (0.92 - 1.21) | 4.81E-01 |  | 1.01 (0.91 - 1.12) | 8.69E-01 |
|  | rs8103262 |  | 1.11 (1.02 - 1.21) | 1.97E-02 |  | 1.06 (0.93 - 1.21) | 3.92E-01 |  | 1.01 (0.91 - 1.12) | 8.80E-01 |
|  | rs867772 |  | 1.11 (1.02 - 1.21) | 2.14E-02 |  | 1.05 (0.92 - 1.21) | 4.52E-01 |  | 1.00 (0.90 - 1.12) | 9.37E-01 |
|  | rs960596 |  | 1.11 (1.01 - 1.21) | 2.18E-02 |  | 1.05 (0.92 - 1.21) | 4.74E-01 |  | 1.00 (0.90 - 1.11) | 9.91E-01 |
|  | rs964184 |  | 1.11 (1.02 - 1.21) | 1.86E-02 |  | 1.05 (0.91 - 1.21) | 5.07E-01 |  | 1.01 (0.91 - 1.12) | 8.64E-01 |
|  | rs9668081 |  | 1.11 (1.01 - 1.20) | 2.31E-02 |  | 1.05 (0.92 - 1.21) | 4.74E-01 |  | 1.00 (0.90 - 1.12) | 9.39E-01 |
| ^a^The leave one SNP out analysis was only performed on micronutrients containing >2 SNP.  ^b^The odds ratios (OR) correspond to a 1 standard deviation increase in the concentration of the micronutrient. | | | | | | | | | | |
