# Supplementary figures and images for: Circulating levels of micronutrients and risk of infections: a Mendelian randomization study
Source: BMC Med. 2023 Mar 8;21:84. doi: 10.1186/s12916-023-02780-3 (PMC9993583; doi:10.1186/s12916-023-02780-3)

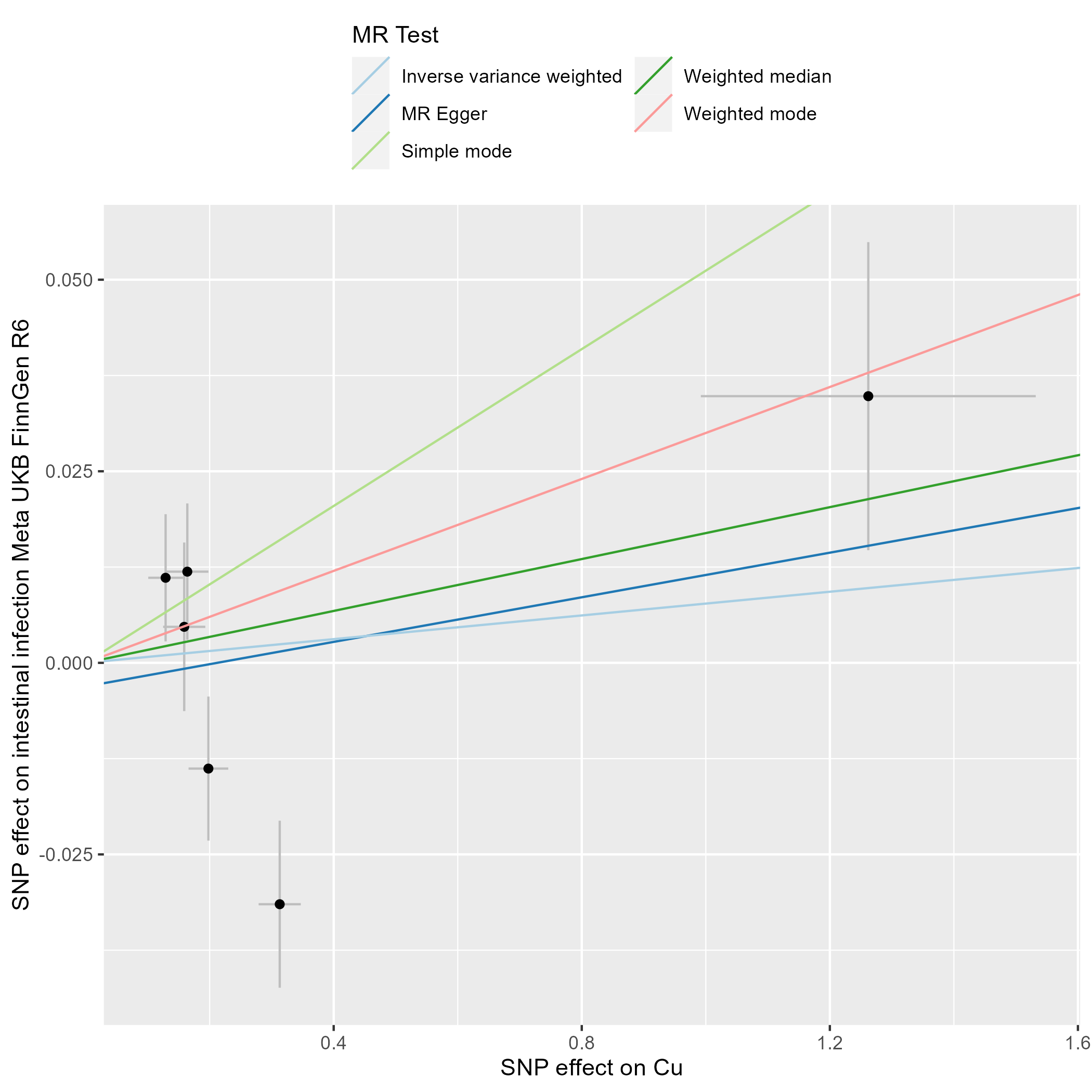

Supplement: Supplementary file 2 — Additional file 2: Additional Text – Exposure GWAS cohorts. Fig. S1. Scatter plot of secondary MR analysis of copper as risk factors on the risk of gastrointestinal infections. Table S2. ICD-10 codes for gastrointestinal infections in UK Biobank and FinnGen R6. Table S3. ICD-10 codes for pneumonia in UK Biobank, and FinnGen. Table S4. ICD-10 codes for urinary tract infection UK Biobank, and FinnGen. Table S6. Power calculations. Table S7. Genetic variants used as exposure for Mendelian randomization analyses. Table S8. Main mendelian randomization analyses of micronutrients as risk factors on the risk of gastrointestinal infections. Table S9. Main mendelian randomization analyses of micronutrients as risk factors on the risk of pneumonia. Table S10. Main mendelian randomization analyses of micronutrients as risk factors on the risk of urinary tract infections. Table S11. Secondary mendelian randomization analyses of micronutrients as risk factors on the risk of gastrointestinal infections, pneumonia and urinary tract infections suggestive-significant genetic instruments. Table S12. Secondary mendelian randomization analyses of copper as risk factors on the risk of gastrointestinal infections, where rs12582659 was removed. Table S21. IVW MR regression results for the leave one SNP out analysis in the Mendelian randomization analyses of micronutrients. [file 12916_2023_2780_MOESM2_ESM.zip › Additional file 2 Fig. S1_ClusterPlot_BMED-D-22-02781R1R2.png]
